# Genotype-phenotype correlations of marfan syndrome and related fibrillinopathies: Phenomenon and molecular relevance

**DOI:** 10.3389/fgene.2022.943083

**Published:** 2022-08-16

**Authors:** Ze-Xu Chen, Wan-Nan Jia, Yong-Xiang Jiang

**Affiliations:** ^1^ Eye Institute and Department of Ophthalmology, Eye & ENT Hospital, Fudan University, Shanghai, China; ^2^ NHC Key Laboratory of Myopia (Fudan University); Key Laboratory of Myopia, Chinese Academy of Medical Sciences, Shanghai, China; ^3^ Shanghai Key Laboratory of Visual Impairment and Restoration, Shanghai, China

**Keywords:** FBN1, type I fibrillinopathy, genotype-phenotype correlation, dominant negative effect, haploinsufficiency

## Abstract

Marfan syndrome (MFS, OMIM: 154700) is a heritable multisystemic disease characterized by a wide range of clinical manifestations. The underlying molecular defect is caused by variants in the *FBN1*. Meanwhile, *FBN1* variants are also detected in a spectrum of connective tissue disorders collectively termed as ‘type I fibrillinopathies’. A multitude of *FBN1* variants is reported and most of them are unique in each pedigree. Although MFS is being considered a monogenic disorder, it is speculated that the allelic heterogeneity of *FBN1* variants contributes to various manifestations, distinct prognoses, and differential responses to the therapies in affected patients. Significant progress in the genotype–phenotype correlations of MFS have emerged in the last 20 years, though, some of the associations were still in debate. This review aims to update the recent advances in the genotype-phenotype correlations of MFS and related fibrillinopathies. The molecular bases and pathological mechanisms are summarized for better support of the observed correlations. Other factors contributing to the phenotype heterogeneity and future research directions were also discussed. Dissecting the genotype-phenotype correlation of *FBN1* variants and related disorders will provide valuable information in risk stratification, prognosis, and choice of therapy.

## Introduction

Marfan syndrome (MFS, OMIM: 154700) is an autosomal dominant connective tissue disorder, characterized by ectopia lentis (EL), aortic dilation, and a combination of skeletal features (Judge and Dietz, 2005). In the latest nosology, MFS is exclusively associated with pathogenic variants in the *FBN1* gene, although a diagnosis of MFS is possible in the absence of genetic testing. However, variants in *FBN1* are also associated with a spectrum of phenotypes (Loeys et al., 2010a). The severe end of this clinical continuum is neonatal MFS characterized by early-onset congestive heart failure secondary to progressive valve problems (Hennekam, 2005; Loeys et al., 2010a). Conditions at the mild end include the MASS syndrome (myopia, mitral valve prolapse, borderline and non-progressive aortic root dilatation, skeletal findings and striae), mitral valve prolapse syndrome, and EL syndrome (Faivre et al., 2012). Some patients with pathogenic *FBN1* variants had features that are different from or even opposite to the manifestations of MFS, such as the skin thickening in stiff skin syndrome (SSS, OMIM: 184900), progeroid appearance in marfanoid–progeroid–lipodystrophy syndrome (MFLS, OMIM: 616914), and short extremities in acromelic dysplasia, which include geleophysic dysplasia (GD, OMIM: 231050), acromicric dysplasia (AD, OMIM: 102370), and type II Weill-Marchesani syndrome (WMS2, OMIM: 608328) (Loeys et al., 2010b; Sakai and Keene, 2019; Muthu and Reinhardt, 2020; Yang et al., 2021). Therefore, patients with *FBN1* variants are collectively termed ‘type I fibrillinopathy’ (Hayward and Brock, 1997; Faivre et al., 2008).

**FIGURE 1:**
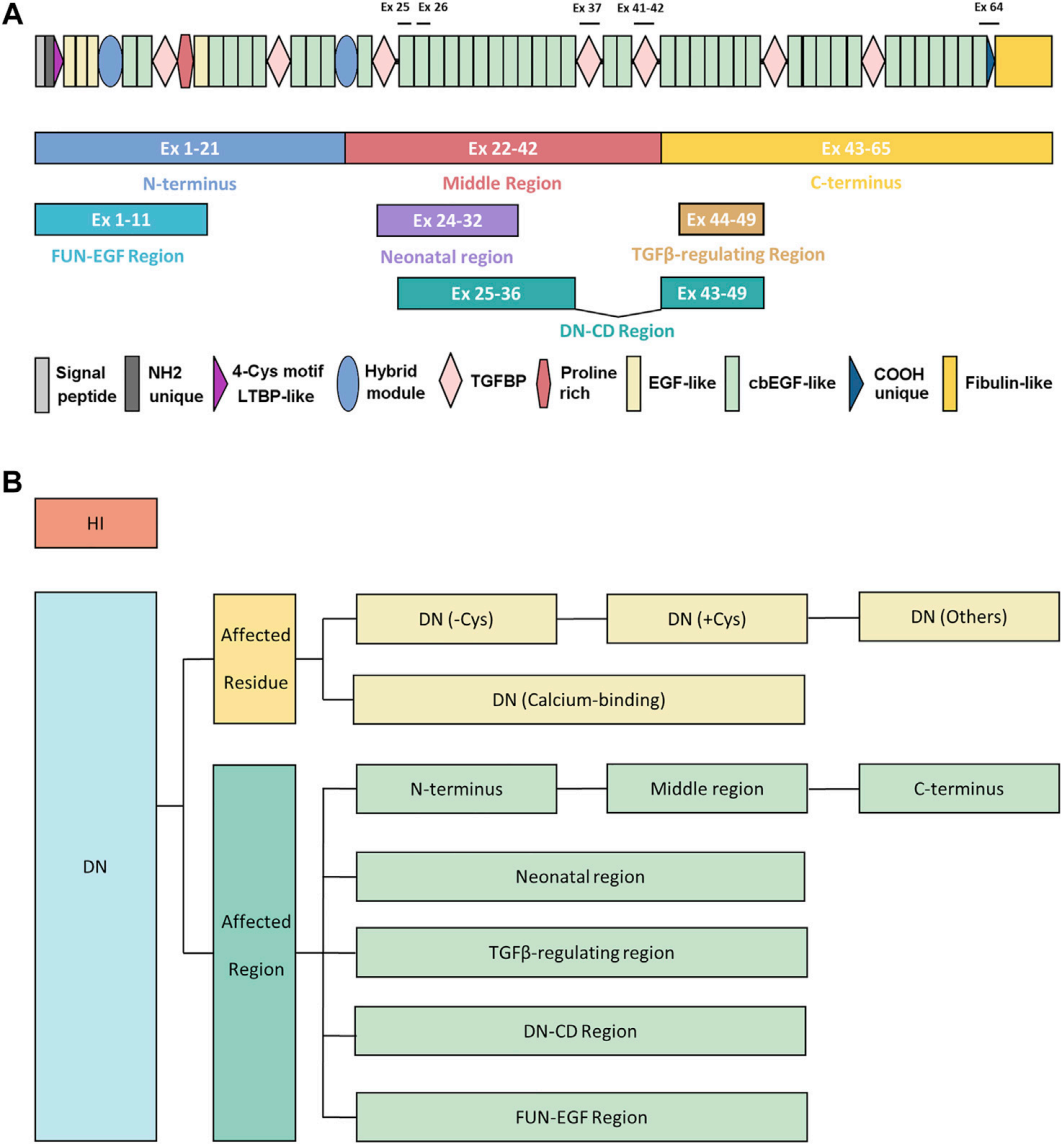
The demonstration of protein architecture and mutation classification strategy of *FBN1* gene. **(A)** The protein architecture of FBN1. The regions in previous genotype-phenotype studies or mechanism studies were shown in colored blocks, including N terminus (exons 1–21), middle region (exons 22–42), C-terminus (exons 43–65), FUN-EGF region (exons 1–11), neonatal region (exons 24–32), TGFβ-regulating region (exons 43–65), DN-CD region (exons 25–36 and exons 43–49). Exons of strong genotype-phenotype correlations were marked, including exon 25 (poorest prognosis), exon 26 (microspherophakia), exon 37 (stiff skin syndrome), exons 41–42 (geleophysic and acromicric dysplasia), exon 64 (marfanoid–progeroid–lipodystrophy syndrome). **(B)** The strategy of mutation classification of *FBN1*. Mutations were first broadly divided into HI and DN groups. DN mutations were further classified according to the affected residue or affected regions. DN mutations were classified as DN (-Cys), DN (+Cys), and DN (Calcium-binding), and DN (Others) based on the affected residues. The classification of affected regions was demonstrated in **(A)**. DN, dominant-negative effect; DN (-Cys), DN mutations eliminating cysteine; DN (+Cys), DN mutations creating cysteine; DN (Calcium-binding), DN mutations affecting conserved calcium-binding motif; DN (Others), DN mutations not belonging to the above groups; Ex, exon; HI, haploinsufficiency.

Molecular testing of *FBN1* has become an integral part of clinical management in patients with MFS. *FBN1* is positioned at chromosome 15q21.1, which encodes a large glycoprotein consisting of 2871 amino acids, with a predicted molecular mass of 350 kDa (Biggin et al., 2004; Loeys et al., 2004). It comprises 47 epidermal growth factor-like domains (EGF-like), among which 43 are calcium-binding (cb EGF-like), seven transforming growth factor β-binding protein domains (TGFBP), two hybrid domains, a proline-rich domain, a 4-Cys motif LTBP-like domain, an NH2 unique domain, a COOH unique domain, and a fibulin-like domain (Figure 1A) (Du et al., 2021). FBN1 polymerizes into microfibrils which are responsible for the elasticity or force-bearing capacity of connective tissue, such as lens zonule, aortic wall, heart valves, and tubular bones (Shin and Yanagisawa, 2019). To date, more than 3000 FBN1 variants have been recorded including the full spectrum of variant types throughout the 65 coding exons and interspersed introns of the gene (Groth et al., 2017). Approximately 30% of cases are caused by new or spontaneous variants and about 12% of all reported *FBN1* variants are recurrent (Collod-Béroud et al., 2003).

Despite high penetrance, one of the unexplained features of MFS and related fibrillinopathies is the prominent phenotype variation in the timing of onset, tissue distribution, and severity of manifestations. A number of studies have tried to explain the phenotypic diversity by the allelic heterogeneity of *FBN1* variants in the last 20 years. Significant correlations have emerged in characteristics, disease progression, risk stratification, and therapeutic responses, encompassing a full range of phenotypes in multiple systems. In this review, we provide an overview of recent advances in the genotype-phenotype correlations of MFS and related fibrillinopathies, list potential molecular bases or mechanisms, and comment on the future research directions, hoping to present illuminating readings for both clinicians and researchers.

## Genotype and phenotype correlation

**FIGURE 2:**
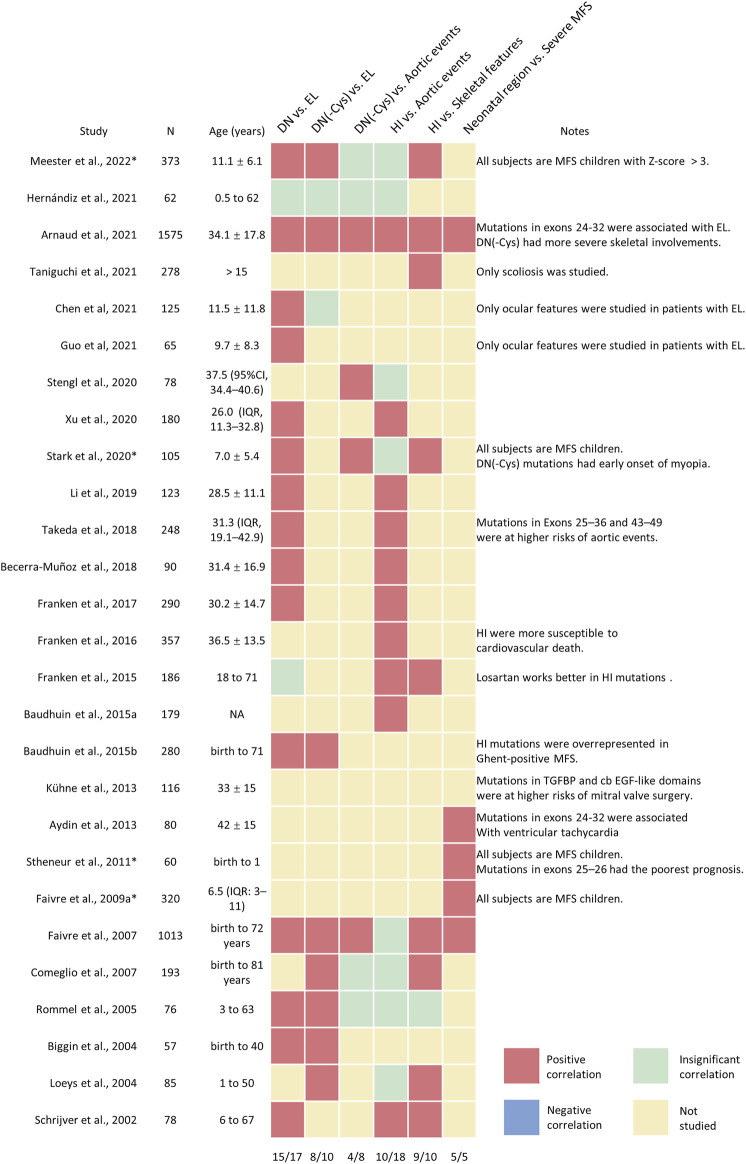
The visualization analysis of genotype-phenotype correlations in patients with MFS and related fibrillinopathies. A heatmap was applied to describe the major conclusions of genotype-correlation studies, with the positive correlations in red, negative correlations in blue; insignificant correlations in green; not-studied correlations in yellow. Minor conclusions or special considerations were shown in the note column. The proportions of positive correlations were shown in the last row.

The first genotype-phenotype correlation came from the clinical observations that variants in patients with neonatal and severe MFS tend to cluster in the exons 24–32 of *FBN1* genes, which were referred to as the neonatal region (Putnam et al., 1996). From then on, more and more studies explore the complexity of the genotype-phenotype correlations. The classification of the *FBN1* variants is summarized in Figure 1B. The genotype-phenotype studies with a sample size of over 50 were summarized in Supplementary Table S1 and visualized in Figure 2. Although no clear-cut manifestations are predictable for a given type of variant, the relative risk for specific organ involvement shows some statistically significant correlations.

## Genotype and clinical manifestations

### Cardiac manifestations

As the major causes of early mortality in MFS, cardiovascular manifestations were the most investigated phenotypes in relation to *FBN1* genotypes. Major cardiovascular involvement includes aortic dilatation and dissection. The dilated aorta generally develops at the aortic root, but the enlargement or dissection of any part of the aorta is possible (Hiratzka et al., 2010). A solid correlation has the potential to optimize the risk stratification of disease monitoring and decision-making in prophylactic surgeries. Schrijver et al. initially classified *FBN1* variants into dominant-negative (DN) variants, encompassing missense variants and inframe deletions or insertions, and haploinsufficiency (HI) groups, including nonsense variants and frameshift variants. It was found that ascending aortic dissections were more common in the HI group than that in the DN group (Schrijver et al., 2002). Salvi et al. found that patients with HI variants exhibited a higher degree of arterial stiffness than that with DN variants, which supports the notion that the aortic involvement is more serious in the HI group (Salvi et al., 2018). Later studies confirmed the above conclusions in independent cohorts; however, this correlation became insignificant in several studies (Figure 2). The potential reasons underlying the inconsistent results may be the different definitions of the aortic events, such as aortic dilation, aortic dissection, or prophylactic surgeries, and the different enrolled patients, such as pediatric patients, classical MFS, or atypical ones.

One big issue is the heterogeneity of the *FBN1* variants. The location of nonsense or frameshift variants did not affect the severity of aortic phenotypes (Faivre et al., 2009a; Takeda et al., 2018). However, the variants in the DN group are of considerable heterogeneity. Missense variants affecting the critical structures, such as disulfide-bond forming cysteines and the conserved calcium-binding motif of the tandem cb EGF-like domains were proved to correlate with more severe MFS (Comeglio et al., 2007; Zhang et al., 2021). Thus, further subgrouping of DN variants is an important step to enlighten the real risk loci within the different types of DN variants. Stengl et al. extracted variants eliminating cysteines (-Cys) from the DN variants and combined them with the HI group. It was found that patients with HI or DN (-Cys) variants had a significantly higher aortic involvement rate than DN variants creating cysteines (+Cys) and other DN variants (Others). When it comes to aortic surgery, patients with DN (-Cys) had the highest risks than HI or other DN variants (Stengl et al., 2020). Arnaud et al. and Faivre et al. also found that DN (-Cys) had a higher severity of aortic dissection or surgery than DN (+Cys) and DN (Others) variants in independent cohorts (Faivre et al., 2007; Arnaud et al., 2021a). Aubart et al. divided MFS patients into severe phenotype group and benign phenotype group based on aortic diameter and aortic surgery history and showed more DN (-Cys) and HI variants in the severe phenotype group but more DN (+Cys) variants in the benign phenotype group (Aubart et al., 2018). Comeglio et al. selected DN variants affecting calcium-binding residues in cb EGF-like domains (Calcium-binding) and combined them with DN (-Cys), which were significantly associated with more severe MFS phenotypes (Comeglio et al., 2007).

Another classification strategy is based on the location of the variants (Figures 1A,B). The most well-known example is the neonatal region (exons 24–32), which correlated with globally higher severity and more complete phenotypes of MFS. Faivre et al. revealed that variants in the neonatal region were associated with a higher probability of ascending aortic dilatation, aortic surgery, and shorter survival, even when cases of neonatal MFS were excluded (Faivre et al., 2007). Similar conclusions were also reported by other studies (Faivre et al., 2009b; Arnaud et al., 2021a). Takeda et al. further extended the risk regions to exon 25–36 and 43–49 based on extreme phenotype sampling and verified the conclusions in an independent cohort (Takeda et al., 2018). DN variants affecting cysteine residues and in-frame deletion variants in exons 25–36 and 43–49 (named DN-CD variants) had larger aortic root Z-scores and a 6.3-fold higher risk of aortic events compared with other patients, which was more deleterious than variants within exons 24–32 and comparable to patients in HI group (Takeda et al., 2018). To sum up, DN variants are a heterogeneous group. DN (-Cys) variants and those located in neonatal or DN-CD region have higher risks of developing aortic events.

Besides aortopathy, other cardiac manifestations are also studied in relation to *FBN1* variants. The HI variants, DN (-Cys) variants, DN variants in the neonatal region, and *FBN1* variants located in TGFBP domains or cb EGF-like domains were associated with higher risks for mitral valve surgery (Faivre et al., 2007; Faivre et al., 2009b; Kühne et al., 2013; Arnaud et al., 2021a). Variants in exons 24–32 were associated with ventricular tachycardia (Aydin et al., 2013). Earlier onset of pulmonary artery dilatation was found in MFS children with DN variants (Stark et al., 2020).

### Ocular manifestations

Ectopia lentis (EL), the dislocation of the lens from its physiological position, was first documented as part of MFS by Börger in 1914 (Über zwei, 1914), and has been recognized as a major criterion or cardinal feature when diagnosing MFS. (Loeys et al., 2010a). The prevalence of EL ranges from 33% to 72% (Konradsen and Zetterstrom, 2013; Chandra et al., 2014), thus, a number of studies aim to address why subsets of patients are exempt from developing EL. Contrary to aortic events, EL was more prevalent in patients harboring DN variants (Becerra-Muñoz et al., 2018; Li et al., 2019), especially the DN (-Cys) or DN (+Cys) ones (Faivre et al., 2007; Baudhuin et al., 2015; Meester et al., 2022). Chen et al. and Guo et al. further proved independently that DN variants were associated with a higher degree of EL compared to the HI ones in cohorts of congenital EL (Chen et al., 2021a; Guo et al., 2021). The variants associated with EL also cluster in the N-terminus of the *FBN1* gene (Comeglio et al., 2007; Faivre et al., 2007; Baudhuin et al., 2015; Meester et al., 2022). And patients harboring variant in the neonatal region also had a higher incidence of EL (Faivre et al., 2007). As a special form of EL, microspherophakia is characterized by the globular laxity of the zonules and the lens growth of which is arrested by insufficient mechanical stretching (Kumar et al., 2019). Microspherophakia was found in about 10% of patients with MFS and the surgical management of which is different from other types of EL (Chen et al., 2021b; Chen et al., 2022). Chen et al. revealed that variants in exons 22–42, especially exon 26, had higher risks of combined microspherophakia in patients with MFS (Chen et al., 2021c).

In recent years, more and more studies of the correlation between ocular manifestations and *FBN1* variants have emerged. The axial length (AL) is the distance from the corneal surface to the retinal pigment. Increased AL was associated with myopia and risks for retinal detachment in patients with MFS (Fan et al., 2014), which has been listed as a minor criterion in Ghent 1 nosology (De Paepe et al., 1996). However, the AL of MFS has considerable individual variations, with short AL in about 30% of MFS patients in a full age range (Drolsum et al., 2015; Chen et al., 2021b). Chen et al. showed that variants in the C-terminus (exons 43–65), especially the TGF-β regulating region (exons 44–49), were associated with longer AL (Chen et al., 2021a). Zhang et al. further showed that DN (-Cys) and DN (Calcium-binding) variants in cb EGF-like domains positively contributed to AL elongation (Zhang et al., 2021). The HI variants and variants in the neonatal region were associated with thinner central corneal thickness (Chen et al., 2021a), which probably explained the conflicting observations of the corneal thickness in MFS (Heur et al., 2008; Konradsen et al., 2012). Posterior staphyloma and ciliary body cysts were more frequently observed in patients with variants in the C-terminus (Chen et al., 2021a). Patients with DN variants tend to show higher corneal astigmatism compared to HI variants (Guo et al., 2021). The correlation between *FBN1* variants and ocular features other than EL only received attention in a few studies, which demand further studies for verification.

### Skeletal and other manifestations

Skeletal manifestations were the most prominent features for MFS patients, including higher arm span/height ratio, lower upper/lower segment ratio, arachnodactyly, kyphosis or scoliosis, and classic craniofacial features (Sakai et al., 2016). Most of the genotype-phenotype correlations studied a combination of skeletal features or systemic scores. Patients harboring HI variants or variants in the neonatal region often show more prominent skeletal features than those with DN ones (Schrijver et al., 2002; Comeglio et al., 2007; Arnaud et al., 2021a; Meester et al., 2022). DN (-Cys) variants were also associated with more severe skeletal manifestations than DN (+Cys) and DN (Others) (Faivre et al., 2009b; Arnaud et al., 2021a). Scoliosis is a sideways curvature of the spine if a Cobb’s angle exceeds 20° on radiographs (Sponseller et al., 1995), which is found in 45%–54% of MFS patients (Faivre et al., 2007; Arnaud et al., 2021a). Taniguchi et al. dissect the genotype-phenotype correlations in MFS already having scoliosis and found that HI variants and variants in the neonatal region were associated with severe scoliosis and faster progression (Taniguchi et al., 2021). Higher systematic scores were observed in the HI group than that in the DN group (Stark et al., 2020). Striae, the stretch marks of the skin, appeared more frequently in patients with HI variants (Faivre et al., 2007). and similar correlations were found in dura ectasia, the stretching of the dural sac (Franken et al., 2015). Hernia occurred earlier in the DN group than that in the HI group (Stark et al., 2020). All in all, like cardiovascular manifestations, skeletal features were more prominent in patients harboring HI variants and those located in the neonatal region.

### Other type I fibrillinopathies

Strong genotype-phenotype correlations exist in some of the subtypes of type I fibrillinopathies. In 2010, Graul-Neumann et al. detected a heterozygotic *FBN1* c.8155_8156del variant in the exon 64 from a 27-year-old patient with congenital lipodystrophy, a progeroid facial appearance, and some signs of MFS (Graul-Neumann et al., 2010). This observation was further supported by independent studies that *FBN1* variants associated with progeroid phenotypes all clustered within exon 64, which is the extreme C-terminus (Goldblatt et al., 2011; Horn and Robinson, 2011; Takenouchi et al., 2013; Jacquinet et al., 2014; Romere et al., 2016; Lin et al., 2020). The term, MFLS, is proposed to recognize the clinically distinct form of fibrillinopathy (Passarge et al., 2016). GD and AD were known as “the mirror image” of MFS, which are characterized by short stature, short extremities, joint limitation, skin thickening, cardiac valvular thickening, and pseudomuscular build (Marzin et al., 2021). *FBN1* variants associated with GD and AD are exclusively distributed in exons 41–42 (Le Goff et al., 2011), indicating that the corresponding region, the fifth TGFBP domain, is associated with short tubular bones and stiff joints. *FBN1* variants causing SSS all cluster within exon 37, corresponding to the fourth TGFBP domain, the disease of which is characterized by joint stiffness and flexion contractures secondary to hard and thick skin (Loeys et al., 2010b). Like GD and AD, WMS2 also belongs to acromelic dysplasia but had a higher prevalence of EL. Most of the *FBN1* variants associated with WMS2 were located in the exons 41–42 (Newell et al., 2017; Cheng et al., 2018), though inframe deletion of exons 9–11 was also reported in a proband of WMS2 (Sengle et al., 2012). Thus, different presentations of *FBN1* variant carriers probably reflect the distinct roles of FBN1 segments.

## Genotype and prognostication

Recent studies not only showed the correlation between *FBN1* variant types and clinical presentation but also revealed that they have prognostic implications on disease progression and survival in MFS patients. The life expectancy of MFS is largely threatened by aortic dissection or rupture, which is the major cause of premature death (Roman and Devereux, 2020). Franken et al. found more rapid aortic dilation in patients with HI than those with DN variants, especially at the aortic root and at the tubular ascending aorta, leading to a 3.3-fold increased risk of death and dissections (Franken et al., 2017). Franken et al. also observed the long-term survival of patients carrying different types of *FBN1* variants. The study demonstrated that individuals with HI variants had a 1.6-fold increased risk for any aortic complication a 2.4-fold increased risk for the combined endpoint comprising death and dissection, and a 2.5-fold increased risk for cardiovascular death compared to patients with a DN variant (Franken et al., 2016). Thus, MFS individuals carrying HI variants should benefit from closer follow-up and more vigorous medical treatment. However, these correlations failed to replicate in an independent cohort of MFS children who had aortic Z-score > 3 (Meester et al., 2022). Thus, the predictive value of *FBN1* variants and disease progression requires further investigations in larger cohorts with appropriate age stratification.

Another set of variants well-known for their poor prognosis is those located in the neonatal region. Though *FBN1* variants located in the neonatal region were neither a sufficient nor necessary condition for developing neonatal MFS, they were overrepresented in MFS patients with severe involvements and were associated with shorter overall survival (Faivre et al., 2007; Faivre et al., 2009b). Faivre et al. conducted in-depth research on variants located in the neonatal region and found that variants in exon 25 were associated with the shortest survival (Faivre et al., 2009a). This observation was further replicated by Stheneur et al. in MFS patients diagnosed before 1 year old (Stheneur et al., 2011). Thus, parents and clinicians caring for young MFS patients with variants in exon 25 should be made aware of the suboptimal prognosis. It is worth noting that patients with a HI located in exons 24–32 rarely displayed a neonatal or severe MFS presentation. The overall MFS phenotype was less severe in HI variants in the neonatal region than in DN variants in the same area (Faivre et al., 2009a). In summary, individuals with DN variants in exons 24–32 and especially exon 25 generally had a poorer prognosis than other variants.

## Genotype and medication

Prophylactic usage of β-blockers is recommended for MFS patients to ameliorate aortic dilation by reducing stress on the aorta (Hiratzka et al., 2010; Erbel et al., 2014). Animal models of Marfan syndrome showed promising outcomes for losartan, an angiotensin II type 1 receptor blocker, as a potential therapy to attenuate aortic dilation (Habashi et al., 2006; Habashi et al., 2011). However, the beneficial effect of losartan was controversial and inconsistent among clinical studies (Groenink et al., 2013; Lacro et al., 2014; Milleron et al., 2015; van Andel et al., 2020). Franken et al. showed that losartan reduced aortic root dilatation rate significantly in adult MFS patients from the HI group instead of the DN group (Franken et al., 2015). Meester et al. conducted a similar clinical trial in MFS children with the Z-score > 3. However, the curative effects of atenolol or losartan did not differ much in patients with DN variants or HI variants (Meester et al., 2022). Thus, it warrants further investigation whether the conflicting results come from the age difference or other potential confounding factors within the two studies. Den Hartog et al. observed that patients with an HI variant showed improvement in biventricular end diastolic volume and stroke volume upon losartan treatment independent of changes in blood pressure, which is not found in DN variant carriers (den Hartog et al., 2016). Despite some inconsistent observations, these studies provide novel insights into the personalized medication for MFS patients based on genetic background.

## Molecular relevance

The correlation between the *FBN1* variant and diverse presentations is not a coincidence but has its molecular relevance. FBN1 is a large glycoprotein with complex multidomain structures and plays different roles in multiple tissues. Thus, the mutation effects are not only complicated by the variant type but also involve the affected loci, tissue diversity, and associated proteins. It is worth noting that most of the experimental evidence is indirect and should be considered regarding the limitations. The underlying mechanism warrants more studies.

### Composition and conformation diversity

The composition of FBN1 microfibril is tissue-dependent. Microfibrils are subgrouped into three categories depending on the association of elastin, including fibers that are elastin-rich microfibril bundles (ERMB), elastin-free microfibril bundles (EFMB), and a continuum intermediate phase (Gawlik, 1965). FBN1 plays a crucial role in elastogenesis, acting as an organized network for the soluble precursor of elastin (tropoelastin). Tropoelastin molecules are deposited extracellularly onto the FBN1 scaffold and are subsequently processed by the lysyl oxidase enzyme for the formation of cross-links (Shin and Yanagisawa, 2019). The organization of elastin-associating microfibrils also differs in organs, which suits the functional integrity of the tissue. The elastic fibers form fenestrated concentric rings that support tissue compliance in the thoracic aorta while organizing as a loose meshwork in the skin, contributing to the pliability of the skin (Ramirez and Sakai, 2010). At the dermal-epidermal junction, the fibers run parallel to the epidermis with turn-offs coursing anchoring the deeper elastic fibers to the basement membrane (Sakai et al., 2016). ERMBs are more elastic in nature and are enriched in the tunica media of the aorta (Shin and Yanagisawa, 2019). The stiffness increases in the aorta of MFS and the pathological findings include elastic lamellae degradation and focal cystic medial necrosis (Nataatmadja et al., 2003; Salvi et al., 2018). There are more EFMBs in tissue where strength and rigidity are needed, including lens zonules and periodontal ligaments. The zonule is a radially oriented apparatus suspending the lens at the optic center and exerting force from the ciliary body in accommodation (Bassnett, 2020). Electron microscopy studies revealed fragmented, wavy, disorientated zonule fibers in eyes of MFS (Farnsworth et al., 1977; Pessier and Potter, 1996). Besides elastin, the presence of some microfibril-associated proteins also differs in organs, including Fibulin-2 (Reinhardt et al., 1996), Perlecan (Tiedemann et al., 2005), and LTBP2 (Hirani et al., 2007). Together, the complex roles of FBN1 and associated proteins in different tissue probably shed light on the organ selectivity of certain *FBN1* variants.

There are also ultrastructure differences of microfibrils, which may correlate with the unique function in specific tissues. Though the exact packing models are still in debate, microfibrils display a typical “beads-on-a-string” appearance with uniform diameters (10–12 nm) and lengths (160 nm) (Cain et al., 2006; Jensen et al., 2012). Eckersley et al. compared the microfibrils isolated from the human eye and those in the skin and found that microfibrils differed in bead morphology and regional proteolytic susceptibility (Eckersley et al., 2018). Specifically, microfibrils in the eye had a higher central bead height and more lytic peptides between the cb EGF-like domains 38–43 than that of the skin, while the inter-bead periodicity was similar (Eckersley et al., 2018). However, only minor differences were observed between bovine adult aorta- and ciliary zonule-derived microfibrils (Lu et al., 2006). To sum up, the above evidence provides fundamental insights into the tissue-specific functions of microfibril but more studies were demanded to further clarify their relationship with the observed genotype-phenotype correlations.

### Mutation effects

At the molecular level, *FBN1* variants are mainly associated with two variant effects: 1) DN effect and 2) HI effect. DN effect generally happens in missense and inframe variants when the incorporation of mutated FBN1 monomers impairs polymerization, thereby creating structurally inferior microfibrils. About 80% of individuals with cysteine substitutions had normal levels of FBN1 synthesis but the secretion was significantly delayed (Schrijver et al., 1999). Experiments showed that both the newly synthesized FBN1 of the normal and mutated allele was retained intracellularly, consistent with the DN effects (Schrijver et al., 1999). The HI effect leads to the degradation of mutant mRNA by the nonsense-mediated decay system, commonly seen in frameshift, nonsense, and splicing variants. In the majority of samples with frameshift or nonsense variants, synthesis of normal FBN1 at protein level measured by pulse-chase analysis of cultured fibroblasts was around 50% of control levels (Schrijver et al., 2002). This decrease leads to the enhanced activation of TGF-β signaling, which results in increased apoptosis, disordered arrangement of smooth muscle cells, and impaired biochemical properties (Granata et al., 2017). Inhibition of TGFβ signaling by neutralizing antibody or losartan, which is an upstream regulator of TGFβ, is effective in preventing aortic aneurysms in *Fbn1* mutant mice (Habashi et al., 2011). However, the association between TGFβ signaling and the pathogenesis of MFS is complicated. The knock-out of TGF-β2 or its receptor has been shown to facilitate aneurysm formation (Lindsay et al., 2012; Wei et al., 2017). And the neutralizing antibody of TGF-β was found to increase the aortic dissection death in another mice model of MFS which was delayed but not prevented by losartan (Cook et al., 2015). Meanwhile, the activation of TGF-β signaling seems to have minimal effects on the microfibril in lens zonules, since variants of genes in TGF‐β signaling could lead to Loeys‐Dietz syndrome with Marfanoid phenotypes in the absence of EL (Meester et al., 2017).

Several studies were devoted to revealing the mechanism differences underlying the HI and DN variants. Xu et al. confirmed the cardinal pathological findings in the aorta of MFS but found that elastic fibers and smooth muscle cells were sparser in patients with HI variants than those observed in patients with DN variants (Xu et al., 2020). Verhagen et al. compared the transcriptomic and proteomic profiles of the aorta aneurysm between HI and DN variants and observed impaired mitochondrial respiration only in HI variants, indicating the critical role of mitochondrial dysfunction in the pathogenesis of aortic aneurysm (Verhagen et al., 2021). Burger et al. revealed reduced SMA expression, decreased pSMAD2/SMAD2 ratio, and impaired transdifferentiation potential upon TGFβ stimulation in the skin fibroblast of DN variants rather than HI variants (Burger et al., 2021). However, this discrepancy is of uncertain significance since the obtained vascular smooth muscle cell-like cells from the transdifferentiation exhibited comparable contractility and migration capacity between the two groups (Burger et al., 2021). However, these studies only studied limited cell types from a small number of patients within DN or HI groups. Thus, more evidence is needed to elucidate the discrepancy underlying DN and HI variants.

However, the ultimate effect of an *FBN1* variant at the protein level is hard to predict on the basis of sequence information alone. *FBN1* variants with premature termination codons might escape nonsense-mediated decay in specific circumstances and produce a truncated protein exerting the DN effect. Alternatively, predicted DN variants can produce a protein that gets trapped in the endoplasmic reticulum and thus leads to diminished FBN1 deposition in the extracellular matrix (Whiteman and Handford, 2003). Moreover, how to classify splicing variants of *FBN1* is another big issue. Previous studies classified splicing variants as HI variants, DN variants, or an independent group. Some of them perform *in silico* prediction or functional experiments, the others excluded splicing variants from analysis (Supplementary Table S2). Though most splicing variants of *FBN1* were predicted to be inframe, the stability of the mutated protein is severely impaired, which showed a similar effect as HI (Liu et al., 2001). Some splicing variants also had multiple outcomes, which further complicated the evaluation (Hu et al., 2022). Cryptic splice sites in the coding sequences also demand more attention in case of misclassification of splicing variants into the DN group, especially for genes with multiple exon-intron junctions like *FBN1* (Sadusky et al., 2004).

### Sequence-specific function

Patients with nonsense or frameshift variants seemed to have phenotypes independent of termination sites (Faivre et al., 2009a; Takeda et al., 2018), probably because of a similar effect on *FBN1* degradation (Lykke-Andersen and Jensen, 2015). In contrast, patients with DN variants display a broad spectrum of presentations, indicating that the coding regions affected by DN variants are probably of diverse functions. FBN1 not only serves mechanical roles but is also involved in cell signal pathways which are mediated by specific sequences. Thus, variants in different regions of *FBN1* are likely to have diverse functions.

The N-terminus of FBN1 serves as an important platform to interact with a multitude of extracellular components. Thomson et al. summarized that the most FBN1-binding proteins interact with FBN1 through the N-terminus (Thomson et al., 2019), including both the extracellular architectures, such as ADAMTS6 (Cain et al., 2016), ADAMTSL2 (Sengle et al., 2012), and CLSTN1 (Cain et al., 2009), as well as signaling modulators encompassing LTBP1 (Ono et al., 2009) and BMP-2, 4, 5, 7, 10 (Wohl et al., 2016). Some of them were associated with congenital EL, such as LTBP2 (Hirani et al., 2007) and ADAMTS10 (Kutz et al., 2011). The N-terminus is also indispensable in the self-assembly of FBN1. Yadin et al. found that the N-terminal sequence to the first three EGF-like domains (FUN-EGF3) interacted with the heparan sulfate near the cell membrane to promote the oligomerization of the C-terminal cb EGF-like domains 41–43, which thereafter competed with the binding of FUN-EGF3 to heparan sulfate, resulting in the regulated end-to-end assembly of FBN1 (Yadin et al., 2013). They also revealed that the Arg62 is the crucial residue for the self-assembly, the missense variant of which is one of the frequent pathogenic variants for congenital EL in a Chinese cohort (Chen et al., 2021c).

FBN1 has a modular structure with a tandem array of EGF-like domains, 43 of which are capable of calcium-binding, the cb EGF-like domains. These domains contain the calcium-binding consensus motif D/N-XD/N-E/Q-Xm-D/N-Xn-Y/F (where m and n are variable), which provides microfibril a rigid rod-like conformation (Downing et al., 1996). An increased protease susceptibility due to reduced calcium affinity is a mechanism reported for missense variants (Booms et al., 2000; Reinhardt et al., 2000). Disulphide bonds formed among the six cysteine residues in EGF-like and cb EGF-like domains, in a C1-C3, C2-C4, and C5-C6 pattern, further contribute to further stabilizing FBN1 (Werner et al., 2000). Variants affecting the cysteines resulted in protein misfolding, leading to defective secretion and stimulation of endoplasmic reticulum stress (Whiteman and Handford, 2003; Suk et al., 2004). These pathological mechanisms emphasize the importance of calcium-binding and disulphide-forming for the structural integrity of FBN1. Thus, the studies above probably explain more severe phenotypes observed in patients with DN (-Cys) and DN (Calcium-binding) variants (Comeglio et al., 2007; Zhang et al., 2021). The neonatal region includes the central longest stretch of 12 cb EGF-like repeats while the newly defined DN-CD region incorporated more arrays of cb EGF-like repeats into the neonatal region (Takeda et al., 2018). Smallridge et al. showed that the 12-13th cb EGF-like domains possess the highest calcium affinity of any other domains in FBN1, which are located in the center of the neonatal region (Smallridge et al., 1999). Thus, the high calcium affinity is crucial for the rigidity of the neonatal region and could probably explain why variants in this region were at risk of severe phenotypes.

Interspersing the cb EGF-like array are the TGFBP domains, which were characterized by four pairs of disulphide bonds in a C1-C3, C2-C6, C4-C7, C5-C8 arrangement with a hydrophobic core. In contrast to the cb EGF-like domains, the TGFBP domains contribute to the flexibility of FBN1 by interacting with flanking cb EGF-like domains (Kielty et al., 2005). A strong correlation is found in TGFBP domains that all *FBN1* variants associated with SSS are all located within the fourth TGFBP domains (exon 37), the unique feature of which is the Arg-Gly-Asn sequence for integrin binding (Bax et al., 2007). Loeys et al. found that *FBN1* variants in exon 37 resulted in excessive and tightly packed microfibril and impaired integrin interaction in SSS, which is distinct from that of MFS (Loeys et al., 2010b). Meanwhile, *FBN1* variants found in individuals with GD and AD are located in the fifth TGFBP domain (exons 41–42) (Loeys et al., 2010b). Jensen et al. compared the synthesis and deposition profile of *FBN1* variants causing SSS, AD, and MFS and concluded that the primary pathology of SSS and AD was the defective cell-surface interactions which were distinct from the defect in FBN1 synthesis or assembly in MFS (Jensen et al., 2015). Therefore, *FBN1* variants in TGFBP domains have distinct mechanisms associated with SSS, GD, or AD.

Increased TGFβ bioavailability has been correlated with *FBN1* variants and contributed to the pathogenesis of aortic dilation. Beyond sequestering the latent complex of the TGF-β, FBN1 also modulates TGF-β signaling through fragments of exons 44–49, which is named as TGF-β regulating sequence. Chaudhry et al. found the recombinant protein fragments encoded by exons 44–49 strongly bind to the N‐terminus of FBN1, which facilitated the displacement of large latent complex and subsequent release of TGF‐β. It is hypothesized that this fragment degraded from FBN1 protein in pathological situations where there is increased proteolysis or inflammation might contribute to the phenotype in Marfan syndrome (Chaudhry et al., 2007). This evidence potentially correlates with the progressive aortic involvement and elongated axial length of the eyeball associated with the variants in this region (Takeda et al., 2018; Chen et al., 2021a). More studies were warranted to further validate the genotype-phenotype correlations in this region.

The extreme C-terminus of FBN1 has a unique role as a circulating hormone, which was discovered by studying the genotype-phenotype correlation. In 2016, Romere et al. identified a novel glucogenic adipokine, asprosin, based on the fact that *FBN1* variants in the exon 64 were associated with atypical ‘MFS’ patients with progeria and lipodystrophia. Asprosin is the C-terminal of pro-FBN1 which is cleaved by the protease furin (Romere et al., 2016). It was secreted by white adipose, triggering the liver glycogenolysis and the release of insulin (Romere et al., 2016). It was further proved to be a centrally-acting orexigenic hormone, which promoted appetite and body weight (Duerrschmid et al., 2017). The discovery of asprosin based on genotype-phenotype correlation studies provides a novel therapeutic target to treat diabetes and obesity.

## Other factors contributing to phenotype

At present, it is not possible to predict the phenotype solely based on *FBN1* variant. Recurrent variants may lead to distinct phenotypes in different probands, let alone the intrafamilial variability, re-enforcing the notion that factors other than the causative variant also contribute to the phenotypic heterogeneity of MFS.

The mutational effects of the *FBN1* gene are modulated at multiple levels from the mRNA transcription and translation to protein multimerization, and from the incorporation into the extracellular matrix to the degradation hereafter. Therefore, the resulting clinical phenotype cannot easily be predicted based on variants at the genomic level alone. One hypothesis is that the phenotype of MFS is modulated by the residual mRNA expression of the unaffected allele. Aubart et al. cultured the skin fibroblasts from a cohort of MFS with HI variants and quantified the expression level of mRNA synthesized from the wild-type allele (Aubart et al., 2015). It was found that a lower residual expression of FBN1 protein accounts for higher risk of EL, pectus abnormality, and aortic dilatation (Aubart et al., 2015). However, it demanded further studies on whether the mRNA expression of *FBN1* could explain the intrafamily variability. (De Backer et al., 2007). Quantitation of FBN1 protein synthesis and matrix deposition seems to be the ultimate method to correlate the MFS phenotype. Aoyama et al. developed ‘Fibrillin phenotyping’ which divided patients with *FBN1* variants into five groups based on the synthesis and deposition of FBN1 protein from the skin fibroblasts (Aoyama et al., 1994). It was found that most missense variants did not affect the synthesis of FBN1 protein while both the missense and frameshift variants can lead to decreased deposition of FBN1. More severe cardiac complications at an earlier age were seen in groups with FBN1 deposition < 35% (Aoyama et al., 1995).

The incorporated microfibrils also interacted with other extracellular proteins. Thus, an alternative explanation for the individual variation is the involvement of modifier genes. Aubart et al. identified co-occurrence of rare variants in *SMAD3* and *COL4A1* and modifier loci containing E*CE1*, *PKG1,* and *SLN* in MFS patients with severe aortic phenotype (Aubart et al., 2018). Common variants of the *COL1A1* had been shown to exert a protective effect on scoliosis while rare variants of *MYH11* were associated with ectopia lentis (Gentilini et al., 2019). Epigenetic factors were found to influence the aortic manifestations and progression in patients with MFS too. Van Andel et al. identified 28 differentially methylated regions (DMPs) associated with aortic diameters, 7 DMPs with aortic diameter growth, and 5 DMPs with aortic events by genome-wide DNA-methylation profiling of peripheral whole-blood samples (van Andel et al., 2021). Meanwhile, around 0.64% of MFS patients carried more than one *FBN1* gene variant which could be overlooked in routine examinations (Arnaud et al., 2017). Somatic mosaicism for a mutant *FBN1* allele was associated with milder manifestations, compared to those of germ-line transmission (Montgomery et al., 1998; Arnaud et al., 2021b).

## Discussion

In monogenic disorders, an established correlation between genotype and phenotype is a premise for predicting prognosis, enabling prophylaxis, and promoting clinical follow-up in affected patients. In the last 20 years, a number of studies tried to dissect the complexity of the genotype-phenotype correlation of MFS and related fibrillinopathies. Though some observations were inconsistent, statistically significant correlations emerged which provided illuminating readings to understand the pathophysiology of MFS. However, most of the reviews focus on the correlations regarding aortic events and lack a summary of potential molecular relevance. (Loeys, 2016; Stengl et al., 2021). In this review, we update the latest genotype-phenotype correlation studies on the full range of the phenotypes of MFS and related fibrillinopathies and provide potential explanations for the observed correlations at the molecular level.

Early studies on genotype-phenotype correlation began with the susceptibility to EL and aortic dilatation (Schrijver et al., 2002; Loeys et al., 2004). Later studies expanded the studied phenotypes to almost all aspects of the MFS manifestations, including the axial length of the eyeball (Chen et al., 2021a), astigmatism of the cornea (Guo et al., 2021), severity of scoliosis (Taniguchi et al., 2021), and incidence of ventricular tachycardia (Aydin et al., 2013). Meanwhile, detailed parameters were applied instead of a broad description of aortic events, such as the aortic root diameters and the aortic stiffness (Franken et al., 2017; Salvi et al., 2018). The long-term survival, disease progression, and response to medication were also explored in relation to the *FBN1* variants (Franken et al., 2015; Franken et al., 2017; den Hartog et al., 2016). The subjects investigated also include various populations, from the Ghent-positive to atypical ones (Faivre et al., 2009c; Baudhuin et al., 2015), and from the adult (Franken et al., 2015; den Hartog et al., 2016) to pediatric patients (Faivre et al., 2009b; Meester et al., 2022). Some correlations even help to establish a new nosology of fibrillinopathies (Loeys et al., 2010b; Passarge et al., 2016; Marzin et al., 2021) and assisted in identifying a novel hormone, i.e., asprosin (Romere et al., 2016; Duerrschmid et al., 2017). In summary, the DN variants are associated with a higher prevalence of EL while severe cardiac and skeletal phenotypes are more common in the HI variant carriers. The DN variants in the neonatal region and DN (-Cys) variants were correlated with globally more severe MFS phenotypes. Thus, molecular testing of *FBN1* variants will play an increasingly important role in the precise medicine of MFS in the near future. However, the correlations regarding ocular and skeletal manifestations received less attention, and qualified research has just emerged in recent years. The disease progression and long-term prognosis of these phenotypes demands more studies. Most studies were cross-sectional and adapted Kaplan-Meier analyses to control the phenotype variation caused by age difference. Nevertheless, the studies of disease progression in long-term follow-up cohorts will provide more valuable insights.

The classifications of *FBN1* variants also evolve as the research goes on (Figures 1A,B). Dividing the *FBN1* variants into DN and HI is commonly seen. It is widely accepted that patients with DN variants have higher risks of EL while those with HI variants are more likely to develop aortic events. However, the associations between HI variants and aortic involvements seem to be less reproductive, even in some large cohorts (Figure 2) (Comeglio et al., 2007; Faivre et al., 2007; Meester et al., 2022) Later researchers came to realize the heterogeneity of DN variants and proposed two subgrouping schemes. The first one was based on the affected amino acid, which divided the DN variants into DN (-Cys), DN (+Cys), or DN (Calcium-binding) variants. The second one classified DN variants based on the affected region, including the N-terminus, C-terminus, and neonatal region. Recent studies suggested that DN (-Cys) variants and variants in the neonatal region were closer to HI variants in nature than other DN variants, (Faivre et al., 2009b; Stengl et al., 2020), while DN (+Cys) variants were associated with fewer aortic events but similarly high incidence of EL when compared to DN (-Cys) (Faivre et al., 2007; Stengl et al., 2020; Arnaud et al., 2021a). A new region was also identified by taking both the protein structure and the variant clusters of extreme phenotypes (Takeda et al., 2018). Thus, it would be interesting to apply these newly developed classification methods to more phenotypes and independent cohorts.

In 2016, Verstraeten et al. proposed that completion of the mechanistic puzzle of MFS might be feasible within the next 10–15 years (Verstraeten et al., 2016). The sparse and disorganized elastin fibers, the overactivation of TGFβ signaling and subsequent tissue remodeling, and the apoptosis of smooth muscle cells were cardinal mechanisms in aortopathy of MFS (Schrenk et al., 2018). Recently, inflammation, endoplasmic reticulum stress, and mitochondrial dysfunction were also reported to contribute to the molecular mechanism (Siegert et al., 2019; Verhagen et al., 2021; Lim et al., 2022). However, it seems that the mechanism underlying the phenotypic diversity of *FBN1* variant carriers demanded more time to elucidate. We summarized the molecular relevance of the observed genotype-phenotype correlations, beyond the widely accepted DN and HI theories. The tissue-specific monomer conformation and polymer composition provided the fundamental insights for the organ selectivity of the same variant while the unique function among certain regions of FBN1 partially explains the variant effects among different variants. However, most of these evidences were indirect and the functional studies of genotype-phenotype correlations of *FBN1* variants are limited. Most of them tested the tissue or fibroblasts derived from the patients or expressed recombinant segments of FBN1 in cultured fibroblasts (Schrijver et al., 1999; Whiteman and Handford, 2003; Burger et al., 2021). Meanwhile, the molecular genotype-phenotype studies were complicated by the large molecular weight and multiple involved cell types. Patient-derived induced pluripotent stem cells (iPSC) have certain potential in tackling the above troubles and are powerful to tools to study genotype-phenotype correlations in certain diseases (Nevin et al., 2017; Kathuria et al., 2020). iPSCs derived from MFS patients were shown to mimic the skeletal and aortic pathology of MFS (Quarto et al., 2012; Granata et al., 2017; Park et al., 2017). Establishing iPSC models of desired types of FBN1 variants and differentiating them into target cells will be helpful in understanding the molecular mechanism underlying genotype-phenotype correlations.

There is a lack of clear-cut genotype-phenotype correlations of MFS so far. Though *FBN1* variants related to some fibrillinopathies, such as GD, AD, SSS, and MFLS exclusively clustered in certain regions, the variants in the same region also led to classical MFS, which is hard to predict without the comprehensive phenotype analysis of the patient. Variants in neonatal region can also occur in patients with mild and atypical MFS (Faivre et al., 2009a; Zhang et al., 2021). Thus, patients with variants of high risks of aortic events should receive more frequent examinations while those carrying less deleterious variants should not be exempted from follow-up, considering the life-long risks of aortic dissection (Gaudry et al., 2021). Researchers have explored other factors contributing to the phenotypes, including *FBN1* expression at mRNA and protein level (Aoyama et al., 1994; Aubart et al., 2015), modifying genes (Aubart et al., 2018), and epigenetic factors (van Andel et al., 2021). Thus, a prediction model of phenotype and prognosis is warranted in the future which should incorporate both genotypes and modifying factors.

Though genetic testing is not mandatory to establish the diagnosis of MFS, with the growing knowledge of genotype-phenotype correlation, *FBN1* sequencing plays an increasingly important role in the clinical management of MFS and related fibrillinopathies in terms of risk stratification, disease monitoring, and personalized medication. Clinical studies of large sample size and long-term follow-up were warranted to settle the inconsistent correlations. More molecular studies would be beneficial which provide more solid evidence for the established correlations.

## References

[B1] Aoyama T. Francke U. Dietz H. C. Furthmayr H. (1994). Quantitative differences in biosynthesis and extracellular deposition of fibrillin in cultured fibroblasts distinguish five groups of Marfan syndrome patients and suggest distinct pathogenetic mechanisms. J. Clin. Investig. 94 (1), 130–137. 10.1172/JCI117298 8040255 PMC296290

[B2] Aoyama T. Francke U. Gasner C. Furthmayr H. (1995). Fibrillin abnormalities and prognosis in Marfan syndrome and related disorders. Am. J. Med. Genet. 58 (2), 169–176. 10.1002/ajmg.1320580216 8533811

[B3] Arnaud P. Hanna N. Aubart M. Leheup B. Dupuis-Girod S. Naudion S. (2017). Homozygous and compound heterozygous mutations in the FBN1 gene: Unexpected findings in molecular diagnosis of marfan syndrome. J. Med. Genet. 54 (2), 100–103. 10.1136/jmedgenet-2016-103996 27582083

[B4] Arnaud P. Milleron O. Hanna N. Ropers J. Ould Ouali N. Affoune A. (2021). Clinical relevance of genotype-phenotype correlations beyond vascular events in a cohort study of 1500 Marfan syndrome patients with FBN1 pathogenic variants. Genet. Med. 23 (7), 1296–1304. 10.1038/s41436-021-01132-x 33731877 PMC8257477

[B5] Arnaud P. Morel H. Milleron O. Gouya L. Francannet C. Da Costa A. (2021). Unsuspected somatic mosaicism for FBN1 gene contributes to Marfan syndrome. Genet. Med. 23 (5), 865–871. 10.1038/s41436-020-01078-6 33495528 PMC8105163

[B6] Aubart M. Gazal S. Arnaud P. Benarroch L. Gross M. S. Buratti J. (2018). Association of modifiers and other genetic factors explain Marfan syndrome clinical variability. Eur. J. Hum. Genet. 26 (12), 1759–1772. 10.1038/s41431-018-0164-9 30087447 PMC6244213

[B7] Aubart M. Gross M. S. Hanna N. Zabot M. T. Sznajder M. Detaint D. (2015). The clinical presentation of Marfan syndrome is modulated by expression of wild-type FBN1 allele. Hum. Mol. Genet. 24 (10), 2764–2770. 10.1093/hmg/ddv037 25652400

[B8] Aydin A. Adsay B. A. Sheikhzadeh S. Keyser B. Rybczynski M. Sondermann C. (2013). Observational cohort study of ventricular arrhythmia in adults with Marfan syndrome caused by FBN1 mutations. Plos One 8 (12), e81281. 10.1371/journal.pone.0081281 24349050 PMC3862481

[B9] Bassnett S. (2020). Zinn's zonule. Prog. Retin Eye Res., 100902. 32980533 10.1016/j.preteyeres.2020.100902 PMC8139560

[B10] Baudhuin L. M. Kotzer K. E. Lagerstedt S. A. (2015). Decreased frequency of FBN1 missense variants in Ghent criteria-positive Marfan syndrome and characterization of novel FBN1 variants. J. Hum. Genet. 60 (5), 241–252. 10.1038/jhg.2015.10 25652356

[B11] Bax D. V. Mahalingam Y. Cain S. Mellody K. Freeman L. Younger K. (2007). Cell adhesion to fibrillin-1: Identification of an arg-gly-asp-dependent synergy region and a heparin-binding site that regulates focal adhesion formation. J. Cell. Sci. 120 (Pt 8), 1383–1392. 10.1242/jcs.003954 17374638

[B12] Becerra-Muñoz V. M. Gómez-Doblas J. J. Porras-Martín C. Such-Martinez M. Crespo-Leiro M. G. Barriales-Villa R. (2018). The importance of genotype-phenotype correlation in the clinical management of Marfan syndrome. Orphanet J. Rare Dis. 13 (1), 16. 10.1186/s13023-017-0754-6 29357934 PMC5778633

[B13] Biggin A. Holman K. Brett M. Bennetts B. Adès L. (2004). Detection of thirty novel FBN1 mutations in patients with Marfan syndrome or a related fibrillinopathy. Hum. Mutat. 23 (1), 99. 10.1002/humu.9207 14695540

[B14] Booms P. Tiecke F. Rosenberg T. Hagemeier C. Robinson P. N. (2000). Differential effect of FBN1 mutations on *in vitro* proteolysis of recombinant fibrillin-1 fragments. Hum. Genet. 107 (3), 216–224. 10.1007/s004390000368 11071382

[B15] Burger J. Bogunovic N. de Wagenaar N. P. Liu H. van Vliet N. IJpma A. (2021). Molecular phenotyping and functional assessment of smooth muscle-like cells with pathogenic variants in aneurysm genes ACTA2, MYH11, SMAD3 and FBN1. Hum. Mol. Genet. 30 (23), 2286–2299. 10.1093/hmg/ddab190 34244757 PMC8600030

[B16] Cain S. A. McGovern A. Small E. Ward L. J. Baldock C. Shuttleworth A. (2009). Defining elastic fiber interactions by molecular fishing: An affinity purification and mass spectrometry approach. Mol. Cell. Proteomics 8 (12), 2715–2732. 10.1074/mcp.M900008-MCP200 19755719 PMC2816023

[B17] Cain S. A. Morgan A. Sherratt M. J. Ball S. G. Shuttleworth C. A. Kielty C. M. (2006). Proteomic analysis of fibrillin-rich microfibrils. Proteomics 6 (1), 111–122. 10.1002/pmic.200401340 16302274

[B18] Cain S. A. Mularczyk E. J. Singh M. Massam-Wu T. Kielty C. M. (2016). ADAMTS-10 and -6 differentially regulate cell-cell junctions and focal adhesions. Sci. Rep. 6, 35956. 10.1038/srep35956 27779234 PMC5078793

[B19] Chandra A. Ekwalla V. Child A. Charteris D. (2014). Prevalence of ectopia lentis and retinal detachment in Marfan syndrome. Acta Ophthalmol. 92 (1), e82–e83. 10.1111/aos.12175 23786577

[B20] Chaudhry S. S. Cain S. A. Morgan A. Dallas S. L. Shuttleworth C. A. Kielty C. M. (2007). Fibrillin-1 regulates the bioavailability of TGFbeta1. J. Cell. Biol. 176 (3), 355–367. 10.1083/jcb.200608167 17242066 PMC2063961

[B21] Chen Z. Chen J. Zhang M. Chen T. H. Zheng J. L. Deng M. (2021b). Analysis of axial length in young patients with marfan syndrome and bilateral ectopia lentis by Z-scores. Ophthalmic Res. 64 (5), 811–819. 10.1159/000517384 34034266

[B22] Chen Z. Chen T. Zhang M. Chen J. Deng M. Zheng J. (2021c). Fibrillin-1 gene mutations in a Chinese cohort with congenital ectopia lentis: Spectrum and genotype–phenotype analysis. Brit J. Ophthalmol., 2021–319084. 10.1136/bjophthalmol-2021-319084 PMC9685704 34281902

[B23] Chen Z. Zhao Z. Sun Y. Jia W. N. Zheng J. L. Chen J. H. (2022). Phacoemulsification combined with supra-capsular and scleral-fixated intraocular lens implantation in microspherophakia: A retrospective comparative study. Front. Med. 9, 869539. 10.3389/fmed.2022.869539 PMC9047048 35492301

[B24] Chen Z. X. Chen T. H. Zhang M. Chen J. H. Lan L. N. Deng M. (2021a). Correlation between FBN1 mutations and ocular features with ectopia lentis in the setting of Marfan syndrome and related fibrillinopathies. Hum. Mutat. 42 (12), 1637–1647. 10.1002/humu.24283 34550612

[B25] Cheng S. W. Luk H. M. Chu Y. Tung Y. L. Kwan E. Y. W. Lo I. F. M. (2018). A report of three families with FBN1-related acromelic dysplasias and review of literature for genotype-phenotype correlation in geleophysic dysplasia. Eur. J. Med. Genet. 61 (4), 219–224. 10.1016/j.ejmg.2017.11.018 29191498

[B26] Collod-Béroud G. Le Bourdelles S. Ades L. Ala-Kokko L. Booms P. Boxer M. (2003). Update of the UMD-FBN1 mutation database and creation of an FBN1 polymorphism database. Hum. Mutat. 22 (3), 199–208. 10.1002/humu.10249 12938084

[B27] Comeglio P. Johnson P. Arno G. Brice G. Evans A. Aragon-Martin J. (2007). The importance of mutation detection in marfan syndrome and marfan-related disorders: Report of 193FBN1 mutations. Hum. Mutat. 28 (9), 928. 10.1002/humu.9505 17657824

[B28] Cook J. R. Clayton N. P. Carta L. Galatioto J. Chiu E. Smaldone S. (2015). Dimorphic effects of transforming growth factor-β signaling during aortic aneurysm progression in mice suggest a combinatorial therapy for Marfan syndrome. Arterioscler. Thromb. Vasc. Biol. 35 (4), 911–917. 10.1161/ATVBAHA.114.305150 25614286 PMC4376614

[B29] De Backer J. Loeys B. Leroy B. Coucke P. Dietz H. De Paepe A. (2007). Utility of molecular analyses in the exploration of extreme intrafamilial variability in the Marfan syndrome. Clin. Genet. 72 (3), 188–198. 10.1111/j.1399-0004.2007.00845.x 17718856

[B145] De Paepe A. Devereux R. B. Dietz H. C. Hennekam R. C. Pyeritz R. E. (1996). Revised diagnostic criteria for the Marfan syndrome. Am. J. Med. Genet. 62, 417–426. 8723076 10.1002/(SICI)1096-8628(19960424)62:4<417::AID-AJMG15>3.0.CO;2-R

[B30] den Hartog A. W. Franken R. van den Berg M. P. Zwinderman A. H. Timmermans J. Scholte A. J. (2016). The effect of losartan therapy on ventricular function in Marfan patients with haploinsufficient or dominant negative FBN1 mutations. Neth. Heart J. 24 (11), 675–681. 10.1007/s12471-016-0905-8 27704402 PMC5065542

[B31] Downing A. K. Knott V. Werner J. M. Cardy C. M. Campbell I. D. Handford P. A. (1996). Solution structure of a pair of calcium-binding epidermal growth factor-like domains: Implications for the marfan syndrome and other genetic disorders. Cell. 85 (4), 597–605. 10.1016/s0092-8674(00)81259-3 8653794

[B32] Drolsum L. Rand-Hendriksen S. Paus B. Geiran O. R. Semb S. O. (2015). Ocular findings in 87 adults with Ghent-1 verified Marfan syndrome. Acta Ophthalmol. 93 (1), 46–53. 10.1111/aos.12448 24853997

[B33] Du Q. Zhang D. Zhuang Y. Xia Q. Wen T. Jia H. (2021). The molecular genetics of marfan syndrome. Int. J. Med. Sci. 18 (13), 2752–2766. 10.7150/ijms.60685 34220303 PMC8241768

[B34] Duerrschmid C. He Y. Wang C. Li C. Bournat J. C. Romere C. (2017). Asprosin is a centrally acting orexigenic hormone. Nat. Med. 23 (12), 1444–1453. 10.1038/nm.4432 29106398 PMC5720914

[B35] Eckersley A. Mellody K. T. Pilkington S. Griffiths C. E. M. Watson R. E. B. O'Cualain R. (2018). Structural and compositional diversity of fibrillin microfibrils in human tissues. J. Biol. Chem. 293 (14), 5117–5133. 10.1074/jbc.RA117.001483 29453284 PMC5892578

[B36] Erbel R. Aboyans V. Boileau C. Bossone E. Bartolomeo R. D. Eggebrecht H. (20142014). 2014 ESC Guidelines on the diagnosis and treatment of aortic diseases: Document covering acute and chronic aortic diseases of the thoracic and abdominal aorta of the adult. The Task Force for the Diagnosis and Treatment of Aortic Diseases of the European Society of Cardiology (ESC). Eur. Heart J. 35 (41), 2873–2926. 10.1093/eurheartj/ehu281 25173340

[B37] Faivre L. Collod-Beroud G. Adès L. Arbustini E. Child A. Callewaert B. L. (2012). The new ghent criteria for marfan syndrome: What do they change? Clin. Genet. 81 (5), 433–442. 10.1111/j.1399-0004.2011.01703.x 21564093

[B38] Faivre L. Collod-Beroud G. Callewaert B. Child A. Binquet C. GautiEr E. (2009). Clinical and mutation-type analysis from an international series of 198 probands with a pathogenic FBN1 exons 24-32 mutation. Eur. J. Hum. Genet. 17 (4), 491–501. 10.1038/ejhg.2008.207 19002209 PMC2734964

[B39] Faivre L. Collod-Beroud G. Callewaert B. Child A. Loeys B. L. Binquet C. (2009). Pathogenic FBN1 mutations in 146 adults not meeting clinical diagnostic criteria for Marfan syndrome: Further delineation of type 1 fibrillinopathies and focus on patients with an isolated major criterion. Am. J. Med. Genet. A 149A (5), 854–860. 10.1002/ajmg.a.32809 19353630

[B40] Faivre L. Collod-Beroud G. Child A. Loeys B. L. Binquet C. Gautier E. (2008). Contribution of molecular analyses in diagnosing marfan syndrome and type I fibrillinopathies: An international study of 1009 probands. J. Med. Genet. 45 (6), 384–390. 10.1136/jmg.2007.056382 18310266

[B41] Faivre L. Collod-Beroud G. Loeys B. L. Child A. Binquet C. GautiEr E. (2007). Effect of mutation type and location on clinical outcome in 1, 013 probands with marfan syndrome or related phenotypes and FBN1 mutations: An international study. Am. J. Hum. Genet. 81 (3), 454–466. 10.1086/520125 17701892 PMC1950837

[B42] Faivre L. Masurel-Paulet A. Collod-Beroud G. Callewaert B. L. Child A. H. Stheneur C. (2009). Clinical and molecular study of 320 children with marfan syndrome and related type I fibrillinopathies in a series of 1009 probands with pathogenic FBN1 mutations. Pediatr. Evanst. 123 (1), 391–398. 10.1542/peds.2008-0703 19117906

[B43] Fan F. Luo Y. Liu X. Lu Y. Zheng T. (2014). Risk factors for postoperative complications in lensectomy–vitrectomy with or without intraocular lens placement in ectopia lentis associated with Marfan syndrome. Br. J. Ophthalmol. 98 (10), 1338–1342. 10.1136/bjophthalmol-2013-304144 24831716

[B44] Farnsworth P. N. Burke P. Dotto M. E. Cinotti A. A. (1977). Ultrastructural abnormalities in a Marfan's syndrome lens. Arch. Ophthalmol. 95 (9), 1601–1606. 10.1001/archopht.1977.04450090123010 901269

[B45] Franken R. den Hartog A. W. Radonic T. Micha D. Maugeri A. van Dijk F. S. (2015). Beneficial outcome of losartan therapy Depends on type of FBN1 mutation in marfan syndrome. Circ. Cardiovasc. Genet. 8 (2), 383–388. 10.1161/CIRCGENETICS.114.000950 25613431

[B46] Franken R. Groenink M. de Waard V. Feenstra H. M. A. Scholte A. J. van den Berg M. P. (2016). Genotype impacts survival in Marfan syndrome. Eur. Heart J. 37 (43), 3285–3290. 10.1093/eurheartj/ehv739 26787436

[B47] Franken R. Teixido-Tura G. Brion M. Forteza A. Rodriguez-Palomares J. Gutierrez L. (2017). Relationship between fibrillin-1 genotype and severity of cardiovascular involvement in Marfan syndrome. Heart 103 (22), 1795–1799. 10.1136/heartjnl-2016-310631 28468757

[B48] Gaudry M. Porto A. Guivier-Curien C. Blanchard A. Bal L. Resseguier N. (2021). Results of a prospective follow-up study after type A aortic dissection repair: A high rate of distal aneurysmal evolution and reinterventions. Eur. J. Cardiothorac. Surg. 61 (1), 152–159. 10.1093/ejcts/ezab317 34355742

[B49] Gawlik Z. (1965). Morphological and morphochemical properties of the elastic system in the motor organ of man. Folia histochem. cytochem. 3 (3), 233–251. 4160090

[B50] Gentilini D. Oliveri A. Fazia T. Pini A. Marelli S. Bernardinelli L. (2019). NGS analysis in Marfan syndrome spectrum: Combination of rare and common genetic variants to improve genotype-phenotype correlation analysis. Plos One 14 (9), e0222506. 10.1371/journal.pone.0222506 31536524 PMC6752800

[B51] Goldblatt J. Hyatt J. Edwards C. Walpole I. (2011). Further evidence for a marfanoid syndrome with neonatal progeroid features and severe generalized lipodystrophy due to frameshift mutations near the 3' end of the FBN1 gene. Am. J. Med. Genet. A 155A (4), 717–720. 10.1002/ajmg.a.33906 21594992

[B52] Granata A. Serrano F. Bernard W. G. McNamara M. Low L. Sastry P. (2017). An iPSC-derived vascular model of Marfan syndrome identifies key mediators of smooth muscle cell death. Nat. Genet. 49 (1), 97–109. 10.1038/ng.3723 27893734

[B53] Graul-Neumann L. M. Kienitz T. Robinson P. N. Baasanjav S. Karow B. Gillessen-Kaesbach G. (2010). Marfan syndrome with neonatal progeroid syndrome-like lipodystrophy associated with a novel frameshift mutation at the 3' terminus of the FBN1-gene. Am. J. Med. Genet. A 152A (11), 2749–2755. 10.1002/ajmg.a.33690 20979188

[B54] Groenink M. den Hartog A. W. Franken R. Radonic T. de Waard V. Timmermans J. (2013). Losartan reduces aortic dilatation rate in adults with marfan syndrome: A randomized controlled trial. Eur. Heart J. 34 (45), 3491–3500. 10.1093/eurheartj/eht334 23999449

[B55] Groth K. A. Von Kodolitsch Y. Kutsche K. Gaustadnes M. Thorsen K. Andersen N. H. (2017). Evaluating the quality of Marfan genotype-phenotype correlations in existing FBN1 databases. Genet. Med. 19 (7), 772–777. 10.1038/gim.2016.181 27906200

[B56] Guo D. Jin G. Zhou Y. Zhang X. Cao Q. Lian Z. (2021). Mutation spectrum and genotype-phenotype correlations in Chinese congenital ectopia lentis patients. Exp. Eye Res. 207, 108570. 10.1016/j.exer.2021.108570 33844962

[B57] Habashi J. P. Doyle J. J. Holm T. M. Aziz H. Schoenhoff F. Bedja D. (2011). Angiotensin II type 2 receptor signaling attenuates aortic aneurysm in mice through ERK antagonism. Science 332 (6027), 361–365. 10.1126/science.1192152 21493863 PMC3097422

[B58] Habashi J. P. Judge D. P. Holm T. M. Cohn R. D. Loeys B. L. Cooper T. K. (2006). Losartan, an AT1 antagonist, prevents aortic aneurysm in a mouse model of Marfan syndrome. Science 312 (5770), 117–121. 10.1126/science.1124287 16601194 PMC1482474

[B59] Hayward C. Brock D. J. (1997). Fibrillin-1 mutations in Marfan syndrome and other type-1 fibrillinopathies. Hum. Mutat. 10 (6), 415–423. 10.1002/(SICI)1098-1004(1997)10:6<415::AID-HUMU1>3.0.CO;2-C 9401003

[B60] Hennekam R. C. (2005). Severe infantile Marfan syndrome versus neonatal Marfan syndrome. Am. J. Med. Genet. A 139 (1), 1. 10.1002/ajmg.a.30979 16222685

[B61] Heur M. Costin B. Crowe S. Grimm R. A. Moran R. Svensson L. G. (2008). The value of keratometry and central corneal thickness measurements in the clinical diagnosis of marfan syndrome. Am. J. Ophthalmol. 145 (6), 997–1001. 10.1016/j.ajo.2008.01.028 18378212

[B62] Hirani R. Hanssen E. Gibson M. A. (2007). LTBP-2 specifically interacts with the amino-terminal region of fibrillin-1 and competes with LTBP-1 for binding to this microfibrillar protein. Matrix Biol. 26 (4), 213–223. 10.1016/j.matbio.2006.12.006 17293099

[B63] Hiratzka L. F. Bakris G. L. Beckman J. A. Bersin R. M. Carr V. F. Casey D. E. (20102010). 2010 ACCF/AHA/AATS/ACR/ASA/SCA/SCAI/SIR/STS/SVM guidelines for the diagnosis and management of patients with thoracic aortic disease: A report of the American college of cardiology foundation/American heart association task force on practice guidelines, American association for thoracic surgery, American college of radiology, American stroke association, society of cardiovascular anesthesiologists, society for cardiovascular angiography and interventions, society of interventional radiology, society of thoracic surgeons, and society for vascular medicine. Circulation 121 (13), e266–e369. 10.1161/CIR.0b013e3181d4739e 20233780

[B64] Horn D. Robinson P. N. (2011). Progeroid facial features and lipodystrophy associated with a novel splice site mutation in the final intron of the FBN1 gene. Am. J. Med. Genet. A 155A (4), 721–724. 10.1002/ajmg.a.33905 21594993

[B65] Hu K. Wan Y. Lee F. Chen J. Wang H. Qu H. (2022). Functional analysis of an intronic FBN1 pathogenic gene variant in a family with marfan syndrome. Front. Genet. 13, 857095. 10.3389/fgene.2022.857095 35547258 PMC9081721

[B66] Jacquinet A. Verloes A. Callewaert B. Coremans C. Coucke P. de Paepe A. (2014). Neonatal progeroid variant of Marfan syndrome with congenital lipodystrophy results from mutations at the 3' end of FBN1 gene. Eur. J. Med. Genet. 57 (5), 230–234. 10.1016/j.ejmg.2014.02.012 24613577

[B67] Jensen S. A. Iqbal S. Bulsiewicz A. Handford P. A. (2015). A microfibril assembly assay identifies different mechanisms of dominance underlying Marfan syndrome, stiff skin syndrome and acromelic dysplasias. Hum. Mol. Genet. 24 (15), 4454–4463. 10.1093/hmg/ddv181 25979247 PMC4492404

[B68] Jensen S. A. Robertson I. B. Handford P. A. (2012). Dissecting the fibrillin microfibril: Structural insights into organization and function. Structure 20 (2), 215–225. 10.1016/j.str.2011.12.008 22325771

[B69] Judge D. P. Dietz H. C. (2005). Marfan's syndrome. Lancet 366 (9501), 1965–1976. 10.1016/S0140-6736(05)67789-6 16325700 PMC1513064

[B70] Kathuria A. Lopez-Lengowski K. Jagtap S. S. McPhie D. Perlis R. H. Cohen B. M. (2020). Transcriptomic landscape and functional characterization of induced pluripotent stem cell-derived cerebral organoids in schizophrenia. JAMA Psychiatry 77 (7), 745–754. 10.1001/jamapsychiatry.2020.0196 32186681 PMC7081156

[B71] Kielty C. M. Sherratt M. J. Marson A. Baldock C. (2005). Fibrillin microfibrils. Adv. Protein Chem. 70, 405–436. 10.1016/S0065-3233(05)70012-7 15837522

[B72] Konradsen T. R. Koivula A. Kugelberg M. Zetterström C. (2012). Corneal curvature, pachymetry, and endothelial cell density in Marfan syndrome. Acta Ophthalmol. 90 (4), 375–379. 10.1111/j.1755-3768.2010.01996.x 21726424

[B73] Konradsen T. R. Zetterstrom C. (2013). A descriptive study of ocular characteristics in Marfan syndrome. Acta Ophthalmol. 91 (8), 751–755. 10.1111/aos.12068 23387925

[B74] Kühne K. Keyser B. Groene E. F. Sheikhzadeh S. Detter C. Lorenzen V. (2013). FBN1 gene mutation characteristics and clinical features for the prediction of mitral valve disease progression. Int. J. Cardiol. 168 (2), 953–959. 10.1016/j.ijcard.2012.10.044 23176764

[B75] Kumar B. Chandler H. L. Plageman T. Reilly M. A. (2019). Lens stretching modulates lens epithelial cell proliferation via YAP regulation. Investig. Ophthalmol. Vis. Sci. 60 (12), 3920–3929. 10.1167/iovs.19-26893 31546253 PMC7043215

[B76] Kutz W. E. Wang L. W. Bader H. L. Majors A. K. Iwata K. Traboulsi E. I. (2011). ADAMTS10 protein interacts with fibrillin-1 and promotes its deposition in extracellular matrix of cultured fibroblasts. J. Biol. Chem. 286 (19), 17156–17167. 10.1074/jbc.M111.231571 21402694 PMC3089559

[B77] Lacro R. V. Dietz H. C. Sleeper L. A. Yetman A. T. Bradley T. J. Colan S. D. (2014). Atenolol versus losartan in children and young adults with Marfan's syndrome. N. Engl. J. Med. 371 (22), 2061–2071. 10.1056/NEJMoa1404731 25405392 PMC4386623

[B78] Le Goff C. Mahaut C. Wang L. W. Allali S. Abhyankar A. Jensen S. (2011). Mutations in the TGFβ binding-protein-like domain 5 of FBN1 are responsible for acromicric and geleophysic dysplasias. Am. J. Hum. Genet. 89 (1), 7–14. 10.1016/j.ajhg.2011.05.012 21683322 PMC3135800

[B79] Li J. Lu C. Wu W. Liu Y. Wang R. Si N. (2019). Application of next-generation sequencing to screen for pathogenic mutations in 123 unrelated Chinese patients with Marfan syndrome or a related disease. Sci. China. Life Sci. 62 (12), 1630–1637. 10.1007/s11427-018-9491-8 31098894

[B80] Lim W. W. Dong J. Ng B. Widjaja A. A. Xie C. Su L. (2022). Inhibition of IL11 signaling reduces aortic pathology in murine marfan syndrome. Circ. Res. 130 (5), 728–740. 10.1161/CIRCRESAHA.121.320381 35135328

[B81] Lin M. Liu Z. Liu G. Zhao S. Li C. Chen W. (2020). Genetic and molecular mechanism for distinct clinical phenotypes conveyed by allelic truncating mutations implicated in FBN1. Mol. Genet. Genomic Med. 8 (1), e1023. 10.1002/mgg3.1023 31774634 PMC6978264

[B82] Lindsay M. E. Schepers D. Bolar N. A. Doyle J. J. Gallo E. Fert-Bober J. (2012). Loss-of-function mutations in TGFB2 cause a syndromic presentation of thoracic aortic aneurysm. Nat. Genet. 44 (8), 922–927. 10.1038/ng.2349 22772368 PMC3616632

[B83] Liu W. Schrijver I. Brenn T. Furthmayr H. Francke U. (2001). Multi-exon deletions of the FBN1 gene in Marfan syndrome. BMC Med. Genet. 2, 11. 10.1186/1471-2350-2-11 11710961 PMC59835

[B84] Loeys B. De Backer J. Van Acker P. WettincK K. Pals G. Nuytinck L. (2004). Comprehensive molecular screening of the FBN1 gene favors locus homogeneity of classical Marfan syndrome. Hum. Mutat. 24 (2), 140–146. 10.1002/humu.20070 15241795

[B85] Loeys B. (2016). The search for genotype/phenotype correlation in marfan syndrome: To be or not to be? Eur. Heart J. 37 (43), 3291–3293. 10.1093/eurheartj/ehw154 27099264

[B86] Loeys B. L. Dietz H. C. Braverman A. C. Callewaert B. L. De Backer J. Devereux R. B. (2010). The revised Ghent nosology for the Marfan syndrome. J. Med. Genet. 47 (7), 476–485. 10.1136/jmg.2009.072785 20591885

[B87] Loeys B. L. Gerber E. E. Riegert-Johnson D. Iqbal S. Whiteman P. McConnell V. (2010). Mutations in fibrillin-1 cause congenital scleroderma: Stiff skin syndrome. Sci. Transl. Med. 2 (23), 23ra20. 10.1126/scitranslmed.3000488 PMC2953713 20375004

[B88] Lu Y. Sherratt M. J. Wang M. C. Baldock C. (2006). Tissue specific differences in fibrillin microfibrils analysed using single particle image analysis. J. Struct. Biol. 155 (2), 285–293. 10.1016/j.jsb.2006.03.021 16697222

[B89] Lykke-Andersen S. Jensen T. H. (2015). Nonsense-mediated mRNA decay: An intricate machinery that shapes transcriptomes. Nat. Rev. Mol. Cell. Biol. 16 (11), 665–677. 10.1038/nrm4063 26397022

[B90] Marzin P. Thierry B. Dancasius A. Cavau A. Michot C. Rondeau S. (2021). Geleophysic and acromicric dysplasias: Natural history, genotype-phenotype correlations, and management guidelines from 38 cases. Genet. Med. 23 (2), 331–340. 10.1038/s41436-020-00994-x 33082559

[B91] Meester J. Peeters S. Van Den Heuvel L. Vandeweyer G. Fransen E. Cappella E. (2022). Molecular characterization and investigation of the role of genetic variation in phenotypic variability and response to treatment in a large pediatric Marfan syndrome cohort. Genet. Med. 24, 1045–1053. 10.1016/j.gim.2021.12.015 35058154 PMC9680912

[B92] Meester J. Verstraeten A. Schepers D. Alaerts M. Van Laer L. Loeys B. L. (2017). Differences in manifestations of Marfan syndrome, Ehlers-Danlos syndrome, and Loeys-Dietz syndrome. Ann. Cardiothorac. Surg. 6 (6), 582–594. 10.21037/acs.2017.11.03 29270370 PMC5721110

[B93] Milleron O. Arnoult F. Ropers J. Aegerter P. Detaint D. Delorme G. (2015). Marfan sartan: A randomized, double-blind, placebo-controlled trial. Eur. Heart J. 36 (32), 2160–2166. 10.1093/eurheartj/ehv151 25935877

[B94] Montgomery R. A. Geraghty M. T. Bull E. Gelb B. D. JohnsonM. McIntosh I. (1998). Multiple molecular mechanisms underlying subdiagnostic variants of Marfan syndrome. Am. J. Hum. Genet. 63 (6), 1703–1711. 10.1086/302144 9837823 PMC1377642

[B95] Muthu M. L. Reinhardt D. P. (2020). Fibrillin-1 and fibrillin-1-derived asprosin in adipose tissue function and metabolic disorders. J. Cell. Commun. Signal. 14 (2), 159–173. 10.1007/s12079-020-00566-3 32279186 PMC7272526

[B96] Nataatmadja M. West M. West J. Summers K. Walker P. Nagata M. (2003). Abnormal extracellular matrix protein transport associated with increased apoptosis of vascular smooth muscle cells in marfan syndrome and bicuspid aortic valve thoracic aortic aneurysm. Circulation 108 (Suppl. 1), I329–I334. 10.1161/01.cir.0000087660.82721.15 12970255

[B97] Nevin Z. S. Factor D. C. Karl R. T. Douvaras P. Laukka J. Windrem M. S. (2017). Modeling the mutational and phenotypic landscapes of pelizaeus-merzbacher disease with human iPSC-derived oligodendrocytes. Am. J. Hum. Genet. 100 (4), 617–634. 10.1016/j.ajhg.2017.03.005 28366443 PMC5384098

[B98] Newell K. Smith W. Ghoshhajra B. Isselbacher E. Lin A. Lindsay M. E. (2017). Cervical artery dissection expands the cardiovascular phenotype in FBN1-related Weill-Marchesani syndrome. Am. J. Med. Genet. A 173 (9), 2551–2556. 10.1002/ajmg.a.38353 28696036

[B99] Ono R. N. Sengle G. Charbonneau N. L. Carlberg V. Bachinger H. P. Sasaki T. (2009). Latent transforming growth factor beta-binding proteins and fibulins compete for fibrillin-1 and exhibit exquisite specificities in binding sites. J. Biol. Chem. 284 (25), 16872–16881. 10.1074/jbc.M809348200 19349279 PMC2719323

[B100] Park J. W. Yan L. Stoddard C. Wang X. Yue Z. Crandall L. (2017). Recapitulating and correcting marfan syndrome in a cellular model. Int. J. Biol. Sci. 13 (5), 588–603. 10.7150/ijbs.19517 28539832 PMC5441176

[B101] Passarge E. Robinson P. N. Graul-Neumann L. M. (2016). Marfanoid-progeroid-lipodystrophy syndrome: A newly recognized fibrillinopathy. Eur. J. Hum. Genet. 24 (9), 1244–1247. 10.1038/ejhg.2016.6 26860060 PMC4989216

[B102] Pessier A. P. Potter K. A. (1996). Ocular pathology in bovine Marfan's syndrome with demonstration of altered fibrillin immunoreactivity in explanted ciliary body cells. Lab. Investig. 75 (1), 87–95. 8683943

[B103] Putnam E. A. Cho M. Zinn A. B. Towbin J. A. Byers P. H. Milewicz D. M. (1996). Delineation of the Marfan phenotype associated with mutations in exons 23-32 of the FBN1 gene. Am. J. Med. Genet. 62 (3), 233–242. 10.1002/(SICI)1096-8628(19960329)62:3<233::AID-AJMG7>3.0.CO;2-U 8882780

[B104] Quarto N. Leonard B. Li S. Marchand M. Anderson E. Behr B. (2012). Skeletogenic phenotype of human Marfan embryonic stem cells faithfully phenocopied by patient-specific induced-pluripotent stem cells. Proc. Natl. Acad. Sci. U. S. A. 109 (1), 215–220. 10.1073/pnas.1113442109 22178754 PMC3252902

[B105] Ramirez F. Sakai L. Y. (2010). Biogenesis and function of fibrillin assemblies. Cell. Tissue Res. 339 (1), 71–82. 10.1007/s00441-009-0822-x 19513754 PMC2819175

[B106] Reinhardt D. P. Ono R. N. Notbohm H. Müller P. K. Bächinger H. P. Sakai L. Y. (2000). Mutations in calcium-binding epidermal growth factor modules render fibrillin-1 susceptible to proteolysis. A potential disease-causing mechanism in Marfan syndrome. J. Biol. Chem. 275 (16), 12339–12345. 10.1074/jbc.275.16.12339 10766875

[B107] Reinhardt D. P. Sasaki T. Dzamba B. J. Keene D. R. Chu M. L. Gohring W. (1996). Fibrillin-1 and fibulin-2 interact and are colocalized in some tissues. J. Biol. Chem. 271 (32), 19489–19496. 10.1074/jbc.271.32.19489 8702639

[B108] Roman M. J. Devereux R. B. (2020). Aortic dissection risk in marfan syndrome. J. Am. Coll. Cardiol. 75 (8), 854–856. 10.1016/j.jacc.2019.12.042 32130919 PMC8208625

[B109] Romere C. Duerrschmid C. Bournat J. Constable P. Jain M. Xia F. (2016). Asprosin, a fasting-induced glucogenic protein hormone. Cell. 165 (3), 566–579. 10.1016/j.cell.2016.02.063 27087445 PMC4852710

[B110] Sadusky T. Newman A. J. Dibb N. J. (2004). Exon junction sequences as cryptic splice sites: Implications for intron origin. Curr. Biol. 14 (6), 505–509. 10.1016/j.cub.2004.02.063 15043816

[B111] Sakai L. Y. Keene D. R. (2019). Fibrillin protein pleiotropy: Acromelic dysplasias. Matrix Biol. 80, 6–13. 10.1016/j.matbio.2018.09.005 30219651

[B112] Sakai L. Y. Keene D. R. Renard M. De Backer J. (2016). FBN1: The disease-causing gene for Marfan syndrome and other genetic disorders. Gene 591 (1), 279–291. 10.1016/j.gene.2016.07.033 27437668 PMC6639799

[B113] Salvi P. Grillo A. Marelli S. Gao L. Salvi L. Viecca M. (2018). Aortic dilatation in marfan syndrome: Role of arterial stiffness and fibrillin-1 variants. J. Hypertens. 36 (1), 77–84. 10.1097/HJH.0000000000001512 29210860

[B114] Schrenk S. Cenzi C. Bertalot T. Conconi M. T. Di Liddo R. (2018). Structural and functional failure of fibrillin-1 in human diseases (Review). Int. J. Mol. Med. 41 (3), 1213–1223. 10.3892/ijmm.2017.3343 29286095

[B115] Schrijver I. Liu W. Brenn T. Furthmayr H. Francke U. (1999). Cysteine substitutions in epidermal growth factor-like domains of fibrillin-1: Distinct effects on biochemical and clinical phenotypes. Am. J. Hum. Genet. 65 (4), 1007–1020. 10.1086/302582 10486319 PMC1288233

[B116] Schrijver I. Liu W. Odom R. Brenn T. Oefner P. Furthmayr H. (2002). Premature termination mutations in FBN1: Distinct effects on differential allelic expression and on protein and clinical phenotypes. Am. J. Hum. Genet. 71 (2), 223–237. 10.1086/341581 12068374 PMC379156

[B117] Sengle G. Tsutsui K. Keene D. R. Tufa S. F. Carlson E. J. Charbonneau N. L. (2012). Microenvironmental regulation by fibrillin-1. PLoS Genet. 8 (1), e1002425. 10.1371/journal.pgen.1002425 22242013 PMC3252277

[B118] Shin S. J. Yanagisawa H. (2019). Recent updates on the molecular network of elastic fiber formation. Essays Biochem. 63 (3), 365–376. 10.1042/EBC20180052 31395654

[B119] Siegert A. M. García D. G. Esteve-Codina A. Navas-Madronal M. Gorbenko Del Blanco D. Alberch J. (2019). A FBN1 3'UTR mutation variant is associated with endoplasmic reticulum stress in aortic aneurysm in Marfan syndrome. Biochim. Biophys. Acta. Mol. Basis Dis. 1865 (1), 107–114. 10.1016/j.bbadis.2018.10.029 30385411

[B120] Smallridge R. S. Whiteman P. Doering K. Handford P. A. Downing A. K. (1999). EGF-like domain calcium affinity modulated by N-terminal domain linkage in human fibrillin-1. J. Mol. Biol. 286 (3), 661–668. 10.1006/jmbi.1998.2536 10024441

[B121] Sponseller P. D. Hobbs W. Riley L. R. Pyeritz R. E. (1995). The thoracolumbar spine in Marfan syndrome. J. Bone Jt. Surg. Am. 77 (6), 867–876. 10.2106/00004623-199506000-00007 7782359

[B122] Stark V. C. Hensen F. Kutsche K. Kortum F. Olfe J. Wiegand P. (2020). Genotype-phenotype correlation in children: The impact of FBN1 variants on pediatric marfan care. Genes. 11 (7), E799. 10.3390/genes11070799 32679894 PMC7397236

[B123] Stengl R. Ágg B. Pólos M. Matyas G. Szabo G. Merkely B. (2021). Potential predictors of severe cardiovascular involvement in marfan syndrome: The emphasized role of genotype-phenotype correlations in improving risk stratification-a literature review. Orphanet J. Rare Dis. 16 (1), 245. 10.1186/s13023-021-01882-6 34059089 PMC8165977

[B124] Stengl R. Bors A. Ágg B. Polos M. Matyas G. Molnar M. J. (2020). Optimising the mutation screening strategy in Marfan syndrome and identifying genotypes with more severe aortic involvement. Orphanet J. Rare Dis. 15 (1), 290. 10.1186/s13023-020-01569-4 33059708 PMC7558671

[B125] Stheneur C. Faivre L. Collod-Béroud G. Gautier E. Binquet C. Bonithon-Kopp C. (2011). Prognosis factors in probands with an FBN1 mutation diagnosed before the age of 1 year. Pediatr. Res. 69 (3), 265–270. 10.1203/PDR.0b013e3182097219 21135753

[B126] Suk J. Y. Jensen S. McGettrick A. Willis A. C. Whiteman P. Redfield C. (2004). Structural consequences of cysteine substitutions C1977Y and C1977R in calcium-binding epidermal growth factor-like domain 30 of human fibrillin-1. J. Biol. Chem. 279 (49), 51258–51265. 10.1074/jbc.M408156200 15371449

[B127] Takeda N. Inuzuka R. Maemura S. Morita H. Nawata K. Fujita D. (2018). Impact of pathogenic FBN1 variant types on the progression of aortic disease in patients with marfan syndrome. Circ. Genom. Precis. Med. 11 (6), e002058. 10.1161/CIRCGEN.117.002058 29848614

[B128] Takenouchi T. Hida M. Sakamoto Y. Torii C. Kosaki R. Takahashi T. (2013). Severe congenital lipodystrophy and a progeroid appearance: Mutation in the penultimate exon of FBN1 causing a recognizable phenotype. Am. J. Med. Genet. A 161A (12), 3057–3062. 10.1002/ajmg.a.36157 24039054

[B129] Taniguchi Y. Takeda N. Inuzuka R. Matsubayashi Y. Kato S. Doi T. (2021). Impact of pathogenic FBN1 variant types on the development of severe scoliosis in patients with Marfan syndrome. J. Med. Genet.–2021-108186. 10.1136/jmedgenet-2021-108186 PMC9811093 34916231

[B130] Thomson J. Singh M. Eckersley A. Cain S. A. Sherratt M. J. Baldock C. (2019). Fibrillin microfibrils and elastic fibre proteins: Functional interactions and extracellular regulation of growth factors. Semin. Cell. Dev. Biol. 89, 109–117. 10.1016/j.semcdb.2018.07.016 30016650 PMC6461133

[B131] Tiedemann K. Sasaki T. Gustafsson E. Gohring W. Batge B. Notbohm H. (2005). Microfibrils at basement membrane zones interact with perlecan via fibrillin-1. J. Biol. Chem. 280 (12), 11404–11412. 10.1074/jbc.M409882200 15657057

[B132] Über zwei F. B. (1914). Über zwei Fälle von Arachnodaktylie. Z Kinderheilkd (12), 161–184.

[B133] van Andel M. M. Groenink M. van den Berg M. P. Timmermans J. Scholte A. J. H. A. Mulder B. J. M. (2021). Genome-wide methylation patterns in Marfan syndrome. Clin. Epigenetics 13 (1), 217. 10.1186/s13148-021-01204-4 34895303 PMC8665617

[B134] van Andel M. M. Indrakusuma R. Jalalzadeh H. Balm R. Timmermans J. Scholte A. J. (2020). Long-term clinical outcomes of losartan in patients with marfan syndrome: Follow-up of the multicentre randomized controlled COMPARE trial. Eur. Heart J. 41 (43), 4181–4187. 10.1093/eurheartj/ehaa377 32548624 PMC7711887

[B135] Verhagen J. Burger J. Bekkers J. A. den Dekker A. T. von der Thusen J. H. Zajec M. (2021). Multi-omics profiling in marfan syndrome: Further insights into the molecular mechanisms involved in aortic disease. Int. J. Mol. Sci. 23 (1), 438. 10.3390/ijms23010438 35008861 PMC8745050

[B136] Verstraeten A. Alaerts M. Van Laer L. Loeys B. (2016). Marfan syndrome and related disorders: 25 Years of gene discovery. Hum. Mutat. 37 (6), 524–531. 10.1002/humu.22977 26919284

[B137] Wei H. Hu J. H. Angelov S. N. Fox K. Yan J. Enstrom R. (2017). Aortopathy in a mouse model of marfan syndrome is not mediated by altered transforming growth factor β signaling. J. Am. Heart Assoc. 6 (1), e004968. 10.1161/JAHA.116.004968 28119285 PMC5523644

[B138] Werner J. M. Knott V. Handford P. A. Campbell I. D. Downing A. K. (2000). Backbone dynamics of a cbEGF domain pair in the presence of calcium. J. Mol. Biol. 296 (4), 1065–1078. 10.1006/jmbi.1999.3513 10686104

[B139] Whiteman P. Handford P. A. (2003). Defective secretion of recombinant fragments of fibrillin-1: Implications of protein misfolding for the pathogenesis of marfan syndrome and related disorders. Hum. Mol. Genet. 12 (7), 727–737. 10.1093/hmg/ddg081 12651868

[B140] Wohl A. P. Troilo H. Collins R. F. Baldock C. Sengle G. (2016). Extracellular regulation of bone morphogenetic protein activity by the microfibril component fibrillin-1. J. Biol. Chem. 291 (24), 12732–12746. 10.1074/jbc.M115.704734 27059954 PMC4933460

[B141] Xu S. Li L. Fu Y. Wang X. Sun H. Wang J. (2020). Increased frequency of FBN1 frameshift and nonsense mutations in Marfan syndrome patients with aortic dissection. Mol. Genet. Genomic Med. 8 (1), e1041. 10.1002/mgg3.1041 31830381 PMC6978253

[B142] Yadin D. A. Robertson I. B. McNaught-Davis J. Evans P. Stoddart D. Handford P. A. (2013). Structure of the fibrillin-1 N-terminal domains suggests that heparan sulfate regulates the early stages of microfibril assembly. Structure 21 (10), 1743–1756. 10.1016/j.str.2013.08.004 24035709 PMC3794157

[B143] Yang G. Y. Huang X. Chen B. J. Xu Z. P. (2021). Weill-Marchesani-like syndrome caused by an FBN1 mutation with low-penetrance. Chin. Med. J. 134 (11), 1359–1361. 10.1097/CM9.0000000000001406 34075901 PMC8183799

[B144] Zhang M. Chen Z. Chen T. Sun X. Jiang Y. (2021). substitution and calcium-binding mutations in FBN1 cbEGF-like domains are associated with severe ocular involvement in patients with congenital ectopia lentis. In Frontiers in cell and developmental biology. (Online first). 10.3389/fcell.2021.816397 PMC8882981 35237611

