# Supplementary material for: Genotype-phenotype correlations of marfan syndrome and related fibrillinopathies: Phenomenon and molecular relevance
Source: Front Genet. 2022 Aug 16;13:943083. doi: 10.3389/fgene.2022.943083 (PMC9514320; doi:10.3389/fgene.2022.943083)
Supplement: Supplementary file 1 [file Table1.docx]

**Table S1. Literature summary of genotype-phenotype correlation studies of Marfan syndrome and related fibrillinopathy.**

| Study | Country | Subjects/  Age | Genetic test | Major conclusions |
| --- | --- | --- | --- | --- |
| Meester et al., 2022* | International | 373 MFS  Children with Z > 3/  11.1 ± 6.1 years | Panel NGS  +MLPA | EL was more prevalent in patients with DN mutations than in those with HI mutations, especially when only the DN (-Cys) or DN (+Cys) mutations were compared with HI mutations. The prevalence of ectopia lentis was higher in the N-terminal region (exons 2-24) and lowest in the C-terminal region (exons 58-66).  A more severe cardiovascular phenotype was associated with exons 24-32 and exons 26 to 49. No difference in aortic root diameters nor aortic root growth progression between the DN and HI groups. The response to atenolol vs losartan was not different between DN and HI mutations either.  Skeletal features, including pectus excavatum and taller stature, were more pronounced in patients harboring HI variants than in patients harboring DN variants. |
| Hernándiz et al., 2021 | Spain | 62/  0.5 to 62 years | Panel NGS  MLPA | No significant phenotypic differences were found between the DN and HI groups.  The HI group tended to have more cases of aortic dissection and occurring at a younger age than the DN group.  Patients with DN (-Cys) seemed to have a higher incidence of EL. |
| Arnaud et al., 2021 | France | 1575/  34.1 ± 17.8 years | Panel NGS  Sanger sequencing | The HI variants were associated with higher lifelong risks of aortic events, shorter life expectancy, higher risks of severe scoliosis, and higher risks for mitral valve surgery but lower risks for EL surgery. Skeletal manifestations were more frequent in the HI group, except for elbow extension limitation. Patients with HI mutations were taller and thinner than patients with DN mutations.  DN (-Cys) had a global higher severity including aortic dissection or surgery, skeletal manifestations, and ophthalmologic phenotypes than DN (+Cys) and DN (Others). The DN (+Cys) had the highest frequency of EL surgery. No significant difference was found when considering DN mutations affecting calcium-binding sequences versus other DN variations.  DN variants located between exons 24 and 32, in particular for DN (−Cys) variants, had globally a more severe impact, concerning cardiovascular events, aortic and mitral valve surgery, EL surgery, and skeletal features. |
| Taniguchi et al., 2021 | Japan | 278/  >15 years | Panel NGS  Sanger sequencing | The HI mutations and mutations in exons 24 and 32 were associated with severe scoliosis and faster progression in Marfan syndrome. |
| Chen et al., 2021 | China | 125/  11.5 ± 11.8 | Panel NGS  MLPA  Sanger sequencing | The HI mutations were associated with less severe EL and a thinner central corneal thickness than DN mutations.  The eyes of patients with mutations in the C‐terminal region had longer AL, a higher incidence of posterior staphyloma, and a higher incidence of ciliary body cysts than those in the middle and N‐terminal regions.  Mutations in the TGF‐β‐regulating sequence had longer AL, larger horizontal corneal diameters, and a higher incidence of posterior staphyloma than those with mutations in other regions.  Mutations in the neonatal region were associated with thinner CCT. |
| Guo et al., 2021 | China | 53/  9.7 ± 8.3 | WES  Sanger sequencing | Probands with DN mutations tend to show high corneal astigmatism and severe EL compared to HI mutations. |
| Stengl et al., 2020 | Hungary | 78/  37.5 (95%CI,  34.4–40.6) | Panel NGS  MLPA | Aortic involvement did not differ significantly among the HI and DN mutations.  The combined group of HI and DN (-Cys) had a significantly higher aortic involvement rate than DN (+Cys) and DN (Others). Patients with DN (-Cys) required significantly more aortic surgeries than HI and DN non-Cys mutations. |
| Xu et al., 2020 | China | 180/  26.0 (IQR, 11.3–32.8) | Panel NGS | The HI mutations were more commonly observed in aortic dissection than in aortic aneurysms than DN mutations.  Patients with DN mutations had a higher rate of EL. |
| Stark et al., 2020* | Germany | 105  Children/  7.0 ± 5.4 years | Panel NGS  Sanger sequencing | DN mutations correlated with a higher prevalence of EL, earlier onset of pulmonary artery dilatation, lower incidence of dura ectasia, and smaller systematic scores than HI mutations. No significant aortic genotype-phenotype correlations for HI mutations in comparison to DN mutations were identified.  Patients with DN (-Cys) and DN (+Cys) more frequently developed aortic dilatation requiring medication), tricuspid valve prolapse, and earlier onset of myopia than DN (Others). Significantly more pectus excavatum in patients with splice variants than in those with missense/in-frame variants, while hernia appeared earlier in the latter. |
| Li et al., 2019 | China | 123/  28.5 ± 11.1 | Panel NGS  MLPA | Patients with a DN mutation had a higher prevalence of EL but lower risks for aortic dissection.  No phenotypic difference between DN (-Cys), DN (+Cys), and DN (Others) was found. |
| Takeda et al., 2018 | Japan | 248/  31.3 (IQR, 19.1–42.9) | Sanger sequencing | Patients with DN mutations were more likely to develop EL. No significant phenotypic differences regarding aortic events were found between the DN and HI groups.  DN variants in exons 25–36 and 43–49 (DN-CD group) had larger aortic root Z-scores and a 6.3-fold higher risk of aortic events compared with other patients, which was comparable to or more deleterious than HI patients. DN-CD variants seemed to be more deleterious than variants within exons 24–32.  The location of HI variants did not affect the probability of severe aortic events, supporting the notion that HI variants were at uniformly high risk for aortic events. |
| Aubart et al., 2018 | France | 102/  >18 years | WES | Extreme phenotype sampling showed more DN (-Cys) and HI variants in the severe phenotype group and more DN(+Cys) variants in the benign phenotype group. |
| Salvi et al., 2018 | Italy | 93/  33.7 (95%CI,  31.2–36.3) | Sanger sequencing | Patients with frameshift mutations had a higher degree of arterial stiffness than that with missense or splicing mutations. |
| Becerra-Muñoz et al., 2018 | Spain | 90/  31.4 ± 16.9 | Panel NGS | Patients with HI mutations presented a higher proportion of aortic events but a lower incidence of EL, compared to patients with DN mutations. |
| Franken et al., 2017 | Spain | 290/  30.2 ± 14.7 years | Sanger sequencing  MLPA | Patients with DN more frequently had EL than patients with HI.  Patients with HI mutations had a more severely affected aortic phenotype, with larger aortic root diameters and a more rapid dilation rate, and tended to have a 3.3-fold increased risk of death and dissections compared with patients with DN mutations. |
| den Hartog et al., 2016 | Netherlands | 163/  38 ± 13 years | Sanger sequencing | Patients with an FBN1 HI variant showed improvement in biventricular end diastolic volume and stroke volume upon losartan treatment, which was not found in DN mutation carriers. |
| Franken et al., 2016 | Netherlands | 357/  36.5 ± 13.5 years | Sanger sequencing  MLPA | Patients with HI mutations had a 1.6-fold increased risk for any aortic complication compared with patients with a DN mutation, a 2.4-fold increased risk for the combined endpoint comprising death and dissection, and a 2.5-fold increased risk for cardiovascular death. |
| Franken et al., 2015 | Netherlands | 186/  18 to 71 years | Sanger sequencing  MLPA | Losartan reduced the aortic root dilatation rate in HI but not in DN patients.  Pectus carinatum, dura ectasia, and skin striae were more commonly seen in HI patients. |
| Baudhuin et al., 2015a | USA | 179/  NA | Sanger sequencing | A higher frequency of HI variants was observed in MFS patients with an aortic event as compared with all other probands.  Aortic events occurred at a younger median age in patients with HI mutations as compared with those with DN variants. |
| Baudhuin et al., 2015b | USA | 280/  birth to 71 years | Sanger sequencing | A lower frequency of DN mutations was observed in the Ghent-positive patients compared with the Ghent-negative patients. The frequency of DN (+Cys) or DN (-Cys) was not statistically significantly different in Ghent-positive patients as compared with Ghent-negative patients.  Higher frequency of DN (+Cys) or DN (-Cys) motions and mutations in N-terminal region (exons 1-15) were found in patients with EL than in those without EL. The HI mutations occurred more frequently in patients without EL. |
| Aalberts et al., 2014 | Netherland | 149/  33.5 ± 11.8 | DHPLC  PCR  MLPA | Left-ventricular dilatation in MFS patients is more often seen in patients with a non-missense mutation and in those patients without an FBN1 mutation. |
| Kühne et al., 2013 | Germany | 116/  33 ± 15 | Sanger sequencing  MLPA | *FBN1* mutations located in TGFBP domains or cb EGF-like domains correlated marginally with mitral valve surgery.  FBN1 mutations did not relate to the progression of mitral valve regurgitation. |
| Aydin et al., 2013 | Germany | 80/  42 ± 15 | Sanger sequencing  MLPA | Mutations in exons 24–32 were associated with ventricular tachycardia. |
| Stheneur et al., 2011* | France | 60  Children/  birth to 1 year | Database | In MFS patients diagnosed before 1 year old, mutations in exons 25–26 were overrepresented and were associated with shorter survival. |
| Faivre et al., 2009a | Multicenter | 320/  6.5 (IQR: 3–11) years | Database | The DN mutations in exons 24–32 were associated with neonatal MFS and classical MFS in pediatric patients. However, patients with HI mutations were less likely to develop neonatal MFS in children. |
| Faivre et al., 2009b* | Multicenter | 198/  NA | Database | Only mutations in exons 24-32 were studied.  EL and mitral insufficiency were more commonly found in patients with DN mutations in exons 24-32 when compared to patients with HI mutations in the same region. A higher frequency of pectus deformity was found in patients with HI mutations in exons 24–32.  Patients with a HI within exons 24–32 rarely displayed a neonatal or severe MFS presentation.  Exon 25 mutations correlated with a younger age at diagnosis of MFS or type I fibrillinopathy, a higher probability of ascending aortic dilatation, mitral regurgitation, valvular surgery, and scoliosis, and a lower chance of survival were all found when compared to patients with a mutation within other exons of the exons 24–32 region. |
| Faivre et al., 2007 | Multicenter | 1013/  birth to 72 years | Database | Patients with a mutation located in the N-terminal region (exons 1-21) had a higher probability of ectopia lentis than the C-terminal region (exons 43-65). A higher probability of EL was found for patients with DN (-Cys) or DN (+Cys). Overall involvements were more prominent in patients with DN (-Cys) than those with DN (+Cys), including ascending aortic dilatation, mitral valve prolapse, arachnodactyly, and joint laxity.  Patients with HI mutations had a more severe skeletal (arachnodactyly, dolichostenomelia, joint hyperlaxity, pectus deformity, high-arched palate, and pes planus) and skin phenotype than did patients with DN mutation. The cumulative probability of a diagnosis of ascending aortic dilatation before or at age 40 years was similar between patients with HI and DN mutations.  Mutations in exons 24–32 were associated with a more severe and complete phenotype, including younger age at diagnosis of type I fibrillinopathy and a higher probability of developing ectopia lentis, ascending aortic dilatation, aortic surgery, mitral valve abnormalities, scoliosis, and shorter survival; the majority of these results were replicated even when cases of neonatal MFS were excluded. |
| Comeglio et al., 2007 | UK | 193/  birth to 81 years | PCR  SSCP  DHPLC | The HI mutations were associated with more prominent major skeletal manifestations but less frequent EL than those with DN (-Cys) or DN (+Cys) mutations, however, the major cardiovascular manifestations did not differ much between the two groups.  The EL group mutations were more frequent in the N-terminal region (exons 1-15) of the gene than the other region, compared with the distribution of the mutations observed in the other groups. Incomplete MFS were frequent at the 3’ end of the gene.  The association of DN (-Cys) with aortic dilation or dissection was not significantly higher when compared with other kinds of mutations. DN (-Cys) and DN (Calcium-binding) mutations were significantly associated with severe MFS phenotypes when compared to other missense mutations. An increased significance was observed if the combination of the above groups and HI mutations was compared to other mutations. |
| Rommel et al., 2005 | Germany | 76/  3 to 63 years | Sanger sequencing | There was a significantly lower incidence of EL in patients who carried HI mutations or DN (Others), as compared to patients with DN (-Cys) mutations or splice site alteration.  Aortic dissection appeared more often in the HI group than DN (-Cys) group, albeit not in a significant fashion. |
| Biggin et al., 2004 | Australia | 57/  birth to 40 years | SSCP  DHPLC | EL was also found to be more prevalent in patients with DN (-Cys) mutations and less prevalent in those with premature termination mutations. |
| Loeys et al., 2004 | Belgium | 85/  birth to 52 years | CSGE/SSCP, DHPLC, Sanger sequencing | The incidence of EL in the MFS patients was higher in DN (-Cys) group than those in the HI group. While major skeletal involvement was more frequent in the HI group. The difference was not significant for the presence of major cardiovascular manifestations between the DN (-Cys) group and those of the HI group. |
| Schrijver et al., 2002 | USA | 78/  6 to 67 years | PCR  SSCA  DHPLC | Patients with HI mutations have more-striking skeletal features, large-joint laxity, and skin phenotype, coupled with a much lower risk of EL and retinal detachment. Ascending aortic dissections were more common in the HI group. |

* The studies only enrolled pediatric patients.

AL, axial length; CSGE, conformation sensitive gel electrophoresis; CCT, central corneal thickness; DHPLC, denaturing high-performance liquid chromatography; DN (-Cys), dominant-negative mutations eliminating cysteine; DN (+Cys), dominant-negative mutations creating cysteine; DN (Others), dominant-negative mutations not involving cysteine; EL, ectopia lentis; HI, haploinsufficiency; MLPA, multiplex ligation-dependent probe amplification; NGS, next-generation sequencing; PCR, polymerase chain reaction; SSCA, single-strand conformation analysis; SSCP, single-strand conformation polymorphism;

**References:**

Aalberts, J.J., van Tintelen, J.P., Meijboom, L.J., Polko, A., Jongbloed, J.D., van der Wal, H., Pals, G., Osinga, J., Timmermans, J., and de Backer, J.*, et al.* (2014). Relation between genotype and left-ventricular dilatation in patients with Marfan syndrome. GENE *534*, 40-43.

Arnaud, P., Milleron, O., Hanna, N., Ropers, J., Ould, O.N., Affoune, A., Langeois, M., Eliahou, L., Arnoult, F., and Renard, P.*, et al.* (2021). Clinical relevance of genotype-phenotype correlations beyond vascular events in a cohort study of 1500 Marfan syndrome patients with FBN1 pathogenic variants. GENET MED *23*, 1296-1304.

Aubart, M., Gazal, S., Arnaud, P., Benarroch, L., Gross, M., Buratti, J., Boland, A., Meyer, V., Zouali, H., and Hanna, N.*, et al.* (2018). Association of modifiers and other genetic factors explain Marfan syndrome clinical variability. EUR J HUM GENET *26*, 1759-1772.

Aydin, A., Adsay, B.A., Sheikhzadeh, S., Keyser, B., Rybczynski, M., Sondermann, C., Detter, C., Steven, D., Robinson, P.N., and Berger, J.*, et al.* (2013). Observational cohort study of ventricular arrhythmia in adults with Marfan syndrome caused by FBN1 mutations. PLOS ONE *8*, e81281.

Baudhuin, L.M., Kotzer, K.E., and Lagerstedt, S.A. (2015a). Increased frequency of FBN1 truncating and splicing variants in Marfan syndrome patients with aortic events. GENET MED *17*, 177-187.

Baudhuin, L.M., Kotzer, K.E., and Lagerstedt, S.A. (2015b). Decreased frequency of FBN1 missense variants in Ghent criteria-positive Marfan syndrome and characterization of novel FBN1 variants. J HUM GENET *60*, 241-252.

Becerra-Muñoz, V.M., Gómez-Doblas, J.J., Porras-Martín, C., Such-Martínez, M., Crespo-Leiro, M.G., Barriales-Villa, R., de Teresa-Galván, E., Jiménez-Navarro, M., and Cabrera-Bueno, F. (2018). The importance of genotype-phenotype correlation in the clinical management of Marfan syndrome. ORPHANET J RARE DIS *13*, 16.

Biggin, A., Holman, K., Brett, M., Bennetts, B., and Adès, L. (2004). Detection of thirty novel FBN1 mutations in patients with Marfan syndrome or a related fibrillinopathy. HUM MUTAT *23*, 99.

Chen, Z.X., Chen, T.H., Zhang, M., Chen, J.H., Lan, L.N., Deng, M., Zheng, J.L., and Jiang, Y.X. (2021). Correlation between FBN1 mutations and ocular features with ectopia lentis in the setting of Marfan syndrome and related fibrillinopathies. HUM MUTAT *42*, 1637-1647.

Comeglio, P., Johnson, P., Arno, G., Brice, G., Evans, A., Aragon-Martin, J., Silva, F.P.D., Kiotsekoglou, A., and Child, A. (2007). The importance of mutation detection in Marfan syndrome and Marfan-related disorders: report of 193FBN1 mutations. HUM MUTAT *28*, 928.

den Hartog, A.W., Franken, R., van den Berg, M.P., Zwinderman, A.H., Timmermans, J., Scholte, A.J., de Waard, V., Spijkerboer, A.M., Pals, G., Mulder, B.J., and Groenink, M. (2016). The effect of losartan therapy on ventricular function in Marfan patients with haploinsufficient or dominant negative FBN1 mutations. NETH HEART J *24*, 675-681.

Faivre, L., Collod-Beroud, G., Callewaert, B., Child, A., Binquet, C., Gautier, E., Loeys, B.L., Arbustini, E., Mayer, K., and Arslan-Kirchner, M.*, et al.* (2009). Clinical and mutation-type analysis from an international series of 198 probands with a pathogenic FBN1 exons 24-32 mutation. EUR J HUM GENET *17*, 491-501.

Faivre, L., Collod-Beroud, G., Loeys, B.L., Child, A., Binquet, C., Gautier, E., Callewaert, B., Arbustini, E., Mayer, K., and Arslan-Kirchner, M.*, et al.* (2007). Effect of mutation type and location on clinical outcome in 1,013 probands with Marfan syndrome or related phenotypes and FBN1 mutations: an international study. AM J HUM GENET *81*, 454-466.

Faivre, L., Masurel-Paulet, A., Collod-Béroud, G., Callewaert, B.L., Child, A.H., Stheneur, C., Binquet, C., Gautier, E., Chevallier, B., and Huet, F.*, et al.* (2009). Clinical and molecular study of 320 children with Marfan syndrome and related type I fibrillinopathies in a series of 1009 probands with pathogenic FBN1 mutations. PEDIATRICS *123*, 391-398.

Franken, R., den Hartog, A.W., Radonic, T., Micha, D., Maugeri, A., van Dijk, F.S., Meijers-Heijboer, H.E., Timmermans, J., Scholte, A.J., and van den Berg, M.P.*, et al.* (2015). Beneficial Outcome of Losartan Therapy Depends on Type of FBN1 Mutation in Marfan Syndrome. Circulation: Cardiovascular Genetics *8*, 383-388.

Franken, R., Groenink, M., de Waard, V., Feenstra, H.M.A., Scholte, A.J., van den Berg, M.P., Pals, G., Zwinderman, A.H., Timmermans, J., and Mulder, B.J.M. (2016). Genotype impacts survival in Marfan syndrome. EUR HEART J *37*, 3285-3290.

Franken, R., Teixido-Tura, G., Brion, M., Forteza, A., Rodriguez-Palomares, J., Gutierrez, L., Garcia, D.D., Pals, G., Mulder, B.J., and Evangelista, A. (2017). Relationship between fibrillin-1 genotype and severity of cardiovascular involvement in Marfan syndrome. HEART *103*, 1795-1799.

Guo, D., Jin, G., Zhou, Y., Zhang, X., Cao, Q., Lian, Z., Guo, Y., and Zheng, D. (2021). Mutation spectrum and genotype-phenotype correlations in Chinese congenital ectopia lentis patients. EXP EYE RES *207*, 108570.

Hernándiz, A., Zúñiga, A., Valera, F., Domingo, D., Ontoria-Oviedo, I., Marí, J.F., Román, J.A., Calvo, I., Insa, B., and Gómez, R.*, et al.* (2021). Genotype FBN1/phenotype relationship in a cohort of patients with Marfan syndrome. CLIN GENET *99*, 269-280.

Kühne, K., Keyser, B., Groene, E.F., Sheikhzadeh, S., Detter, C., Lorenzen, V., Hillebrand, M., Bernhardt, A.M., Hoffmann, B., and Mir, T.S.*, et al.* (2013). FBN1 gene mutation characteristics and clinical features for the prediction of mitral valve disease progression. INT J CARDIOL *168*, 953-959.

Li, J., Lu, C., Wu, W., Liu, Y., Wang, R., Si, N., Meng, X., Zhang, S., and Zhang, X. (2019). Application of next-generation sequencing to screen for pathogenic mutations in 123 unrelated Chinese patients with Marfan syndrome or a related disease. Science China Life Sciences *62*, 1630-1637.

Loeys, B., De Backer, J., Van Acker, P., Wettinck, K., Pals, G., Nuytinck, L., Coucke, P., and De Paepe, A. (2004). Comprehensive molecular screening of the FBN1 gene favors locus homogeneity of classical Marfan syndrome. HUM MUTAT *24*, 140-146.

Meester, J., Peeters, S., Van Den Heuvel, L., Vandeweyer, G., Fransen, E., Cappella, E., Dietz, H.C., Forbus, G., Gelb, B.D., and Goldmuntz, E.*, et al.* (2022). Molecular characterization and investigation of the role of genetic variation in phenotypic variability and response to treatment in a large pediatric Marfan syndrome cohort. GENET MED.

Rommel, K., Karck, M., Haverich, A., von Kodolitsch, Y., Rybczynski, M., Müller, G., Singh, K.K., Schmidtke, J., and Arslan-Kirchner, M. (2005). Identification of 29 novel and nine recurrent fibrillin-1 (FBN1) mutations and genotype-phenotype correlations in 76 patients with Marfan syndrome. HUM MUTAT *26*, 529-539.

Salvi, P., Grillo, A., Marelli, S., Gao, L., Salvi, L., Viecca, M., Di Blasio, A.M., Carretta, R., Pini, A., and Parati, G. (2018). Aortic dilatation in Marfan syndrome: role of arterial stiffness and fibrillin-1 variants. J HYPERTENS *36*, 77-84.

Schrijver, I., Liu, W., Odom, R., Brenn, T., Oefner, P., Furthmayr, H., and Francke, U. (2002). Premature termination mutations in FBN1: distinct effects on differential allelic expression and on protein and clinical phenotypes. AM J HUM GENET *71*, 223-237.

Stark, V.C., Hensen, F., Kutsche, K., Kortüm, F., Olfe, J., Wiegand, P., von Kodolitsch, Y., Kozlik-Feldmann, R., Müller, G.C., and Mir, T.S. (2020). Genotype-Phenotype Correlation in Children: The Impact of FBN1 Variants on Pediatric Marfan Care. Genes (Basel) *11*.

Stengl, R., Bors, A., Ágg, B., Pólos, M., Matyas, G., Molnár, M.J., Fekete, B., Csabán, D., Andrikovics, H., and Merkely, B.*, et al.* (2020). Optimising the mutation screening strategy in Marfan syndrome and identifying genotypes with more severe aortic involvement. ORPHANET J RARE DIS *15*, 290.

Stheneur, C., Faivre, L., Collod-Béroud, G., Gautier, E., Binquet, C., Bonithon-Kopp, C., Claustres, M., Child, A.H., Arbustini, E., and Adès, L.C.*, et al.* (2011). Prognosis factors in probands with an FBN1 mutation diagnosed before the age of 1 year. PEDIATR RES *69*, 265-270.

Takeda, N., Inuzuka, R., Maemura, S., Morita, H., Nawata, K., Fujita, D., Taniguchi, Y., Yamauchi, H., Yagi, H., and Kato, M.*, et al.* (2018). Impact of Pathogenic FBN1 Variant Types on the Progression of Aortic Disease in Patients With Marfan Syndrome. Circ Genom Precis Med *11*, e2058.

Taniguchi, Y., Takeda, N., Inuzuka, R., Matsubayashi, Y., Kato, S., Doi, T., Yagi, H., Yamauchi, H., Ando, M., Oshima, Y., and Tanaka, S. (2021). Impact of pathogenic FBN1 variant types on the development of severe scoliosis in patients with Marfan syndrome. J MED GENET.

Xu, S., Li, L., Fu, Y., Wang, X., Sun, H., Wang, J., Han, L., Wu, Z., Liu, Y., and Zhu, J.*, et al.* (2020). Increased frequency of FBN1 frameshift and nonsense mutations in Marfan syndrome patients with aortic dissection. Mol Genet Genomic Med *8*, e1041.
