# Supplementary material for: Genotype-phenotype correlations of marfan syndrome and related fibrillinopathies: Phenomenon and molecular relevance
Source: Front Genet. 2022 Aug 16;13:943083. doi: 10.3389/fgene.2022.943083 (PMC9514320; doi:10.3389/fgene.2022.943083)
Supplement: Supplementary file 2 [file Table2.docx]

**Table S2. Literature summary of the classification of splicing mutations in the genotype-phenotype correlation studies of Marfan syndrome and related fibrillinopathy**.

| Classification of splicing mutations | Study | Number |
| --- | --- | --- |
| Splicing mutations were classified as HI mutations | Hernándiz et al., 2021; Arnaud et al., 2021; Taniguchi et al., 2021; Guo et al., 2021;  Stengl et al., 2020; Takeda et al., 2018; Aubart et al., 2018; Baudhuin et al., 2015a; Comeglio et al., 2007; | 9 |
| Splicing mutations were classified as DN mutations | Li et al., 2019; | 1 |
| Splicing mutations with unproven effects  were excluded | Meester et al., 2022; Chen et al., 2021; den Hartog et al., 2016; Faivre et al., 2009a;  Faivre et al., 2009b; Faivre et al., 2007 | 6 |
| The mutation effects were predicted by software | Salvi et al., 2018; Franken et al., 2017; Franken et al., 2015 | 3 |
| The mutation effects were validated by functional experiments | Franken et al., 2016; Schrijver et al., 2002 | 2 |
| Splicing mutations were classified as an independent group. | Xu et al., 2020; Stark et al., 2020; Baudhuin et al., 2015b; Kühne et al., 2013;  Aydin et al., 2013; Rommel et al., 2005 | 6 |
| The classification of splicing mutations was not mentioned | Becerra-Muñoz et al., 2018; Aalberts et al., 2014; Stheneur et al., 2011; Loeys et al., 2004  Biggin et al., 2004; | 5 |

DN, dominant-negative; HI, haploinsufficiency.

**Reference:**

Aalberts, J.J., van Tintelen, J.P., Meijboom, L.J., Polko, A., Jongbloed, J.D., van der Wal, H., Pals, G., Osinga, J., Timmermans, J., and de Backer, J.*, et al.* (2014). Relation between genotype and left-ventricular dilatation in patients with Marfan syndrome. GENE *534*, 40-43.

Arnaud, P., Milleron, O., Hanna, N., Ropers, J., Ould, O.N., Affoune, A., Langeois, M., Eliahou, L., Arnoult, F., and Renard, P.*, et al.* (2021). Clinical relevance of genotype-phenotype correlations beyond vascular events in a cohort study of 1500 Marfan syndrome patients with FBN1 pathogenic variants. GENET MED *23*, 1296-1304.

Aubart, M., Gazal, S., Arnaud, P., Benarroch, L., Gross, M., Buratti, J., Boland, A., Meyer, V., Zouali, H., and Hanna, N.*, et al.* (2018). Association of modifiers and other genetic factors explain Marfan syndrome clinical variability. EUR J HUM GENET *26*, 1759-1772.

Aydin, A., Adsay, B.A., Sheikhzadeh, S., Keyser, B., Rybczynski, M., Sondermann, C., Detter, C., Steven, D., Robinson, P.N., and Berger, J.*, et al.* (2013). Observational cohort study of ventricular arrhythmia in adults with Marfan syndrome caused by FBN1 mutations. PLOS ONE *8*, e81281.

Baudhuin, L.M., Kotzer, K.E., and Lagerstedt, S.A. (2015a). Increased frequency of FBN1 truncating and splicing variants in Marfan syndrome patients with aortic events. GENET MED *17*, 177-187.

Baudhuin, L.M., Kotzer, K.E., and Lagerstedt, S.A. (2015b). Decreased frequency of FBN1 missense variants in Ghent criteria-positive Marfan syndrome and characterization of novel FBN1 variants. J HUM GENET *60*, 241-252.

Becerra-Muñoz, V.M., Gómez-Doblas, J.J., Porras-Martín, C., Such-Martínez, M., Crespo-Leiro, M.G., Barriales-Villa, R., de Teresa-Galván, E., Jiménez-Navarro, M., and Cabrera-Bueno, F. (2018). The importance of genotype-phenotype correlation in the clinical management of Marfan syndrome. ORPHANET J RARE DIS *13*, 16.

Biggin, A., Holman, K., Brett, M., Bennetts, B., and Adès, L. (2004). Detection of thirty novel FBN1 mutations in patients with Marfan syndrome or a related fibrillinopathy. HUM MUTAT *23*, 99.

Chen, Z.X., Chen, T.H., Zhang, M., Chen, J.H., Lan, L.N., Deng, M., Zheng, J.L., and Jiang, Y.X. (2021). Correlation between FBN1 mutations and ocular features with ectopia lentis in the setting of Marfan syndrome and related fibrillinopathies. HUM MUTAT *42*, 1637-1647.

Comeglio, P., Johnson, P., Arno, G., Brice, G., Evans, A., Aragon-Martin, J., Silva, F.P.D., Kiotsekoglou, A., and Child, A. (2007). The importance of mutation detection in Marfan syndrome and Marfan-related disorders: report of 193FBN1 mutations. HUM MUTAT *28*, 928.

den Hartog, A.W., Franken, R., van den Berg, M.P., Zwinderman, A.H., Timmermans, J., Scholte, A.J., de Waard, V., Spijkerboer, A.M., Pals, G., Mulder, B.J., and Groenink, M. (2016). The effect of losartan therapy on ventricular function in Marfan patients with haploinsufficient or dominant negative FBN1 mutations. NETH HEART J *24*, 675-681.

Faivre, L., Collod-Beroud, G., Callewaert, B., Child, A., Binquet, C., Gautier, E., Loeys, B.L., Arbustini, E., Mayer, K., and Arslan-Kirchner, M.*, et al.* (2009). Clinical and mutation-type analysis from an international series of 198 probands with a pathogenic FBN1 exons 24-32 mutation. EUR J HUM GENET *17*, 491-501.

Faivre, L., Collod-Beroud, G., Loeys, B.L., Child, A., Binquet, C., Gautier, E., Callewaert, B., Arbustini, E., Mayer, K., and Arslan-Kirchner, M.*, et al.* (2007). Effect of mutation type and location on clinical outcome in 1,013 probands with Marfan syndrome or related phenotypes and FBN1 mutations: an international study. AM J HUM GENET *81*, 454-466.

Faivre, L., Masurel-Paulet, A., Collod-Béroud, G., Callewaert, B.L., Child, A.H., Stheneur, C., Binquet, C., Gautier, E., Chevallier, B., and Huet, F.*, et al.* (2009). Clinical and molecular study of 320 children with Marfan syndrome and related type I fibrillinopathies in a series of 1009 probands with pathogenic FBN1 mutations. PEDIATRICS *123*, 391-398.

Franken, R., den Hartog, A.W., Radonic, T., Micha, D., Maugeri, A., van Dijk, F.S., Meijers-Heijboer, H.E., Timmermans, J., Scholte, A.J., and van den Berg, M.P.*, et al.* (2015). Beneficial Outcome of Losartan Therapy Depends on Type of FBN1 Mutation in Marfan Syndrome. Circulation: Cardiovascular Genetics *8*, 383-388.

Franken, R., Groenink, M., de Waard, V., Feenstra, H.M.A., Scholte, A.J., van den Berg, M.P., Pals, G., Zwinderman, A.H., Timmermans, J., and Mulder, B.J.M. (2016). Genotype impacts survival in Marfan syndrome. EUR HEART J *37*, 3285-3290.

Franken, R., Teixido-Tura, G., Brion, M., Forteza, A., Rodriguez-Palomares, J., Gutierrez, L., Garcia, D.D., Pals, G., Mulder, B.J., and Evangelista, A. (2017). Relationship between fibrillin-1 genotype and severity of cardiovascular involvement in Marfan syndrome. HEART *103*, 1795-1799.

Guo, D., Jin, G., Zhou, Y., Zhang, X., Cao, Q., Lian, Z., Guo, Y., and Zheng, D. (2021). Mutation spectrum and genotype-phenotype correlations in Chinese congenital ectopia lentis patients. EXP EYE RES *207*, 108570.

Hernándiz, A., Zúñiga, A., Valera, F., Domingo, D., Ontoria-Oviedo, I., Marí, J.F., Román, J.A., Calvo, I., Insa, B., and Gómez, R.*, et al.* (2021). Genotype FBN1/phenotype relationship in a cohort of patients with Marfan syndrome. CLIN GENET *99*, 269-280.

Kühne, K., Keyser, B., Groene, E.F., Sheikhzadeh, S., Detter, C., Lorenzen, V., Hillebrand, M., Bernhardt, A.M., Hoffmann, B., and Mir, T.S.*, et al.* (2013). FBN1 gene mutation characteristics and clinical features for the prediction of mitral valve disease progression. INT J CARDIOL *168*, 953-959.

Li, J., Lu, C., Wu, W., Liu, Y., Wang, R., Si, N., Meng, X., Zhang, S., and Zhang, X. (2019). Application of next-generation sequencing to screen for pathogenic mutations in 123 unrelated Chinese patients with Marfan syndrome or a related disease. Science China Life Sciences *62*, 1630-1637.

Loeys, B., De Backer, J., Van Acker, P., Wettinck, K., Pals, G., Nuytinck, L., Coucke, P., and De Paepe, A. (2004). Comprehensive molecular screening of the FBN1 gene favors locus homogeneity of classical Marfan syndrome. HUM MUTAT *24*, 140-146.

Meester, J., Peeters, S., Van Den Heuvel, L., Vandeweyer, G., Fransen, E., Cappella, E., Dietz, H.C., Forbus, G., Gelb, B.D., and Goldmuntz, E.*, et al.* (2022). Molecular characterization and investigation of the role of genetic variation in phenotypic variability and response to treatment in a large pediatric Marfan syndrome cohort. GENET MED.

Rommel, K., Karck, M., Haverich, A., von Kodolitsch, Y., Rybczynski, M., Müller, G., Singh, K.K., Schmidtke, J., and Arslan-Kirchner, M. (2005). Identification of 29 novel and nine recurrent fibrillin-1 (FBN1) mutations and genotype-phenotype correlations in 76 patients with Marfan syndrome. HUM MUTAT *26*, 529-539.

Salvi, P., Grillo, A., Marelli, S., Gao, L., Salvi, L., Viecca, M., Di Blasio, A.M., Carretta, R., Pini, A., and Parati, G. (2018). Aortic dilatation in Marfan syndrome: role of arterial stiffness and fibrillin-1 variants. J HYPERTENS *36*, 77-84.

Schrijver, I., Liu, W., Odom, R., Brenn, T., Oefner, P., Furthmayr, H., and Francke, U. (2002). Premature termination mutations in FBN1: distinct effects on differential allelic expression and on protein and clinical phenotypes. AM J HUM GENET *71*, 223-237.

Stark, V.C., Hensen, F., Kutsche, K., Kortüm, F., Olfe, J., Wiegand, P., von Kodolitsch, Y., Kozlik-Feldmann, R., Müller, G.C., and Mir, T.S. (2020). Genotype-Phenotype Correlation in Children: The Impact of FBN1 Variants on Pediatric Marfan Care. Genes (Basel) *11*.

Stengl, R., Bors, A., Ágg, B., Pólos, M., Matyas, G., Molnár, M.J., Fekete, B., Csabán, D., Andrikovics, H., and Merkely, B.*, et al.* (2020). Optimising the mutation screening strategy in Marfan syndrome and identifying genotypes with more severe aortic involvement. ORPHANET J RARE DIS *15*, 290.

Stheneur, C., Faivre, L., Collod-Béroud, G., Gautier, E., Binquet, C., Bonithon-Kopp, C., Claustres, M., Child, A.H., Arbustini, E., and Adès, L.C.*, et al.* (2011). Prognosis factors in probands with an FBN1 mutation diagnosed before the age of 1 year. PEDIATR RES *69*, 265-270.

Takeda, N., Inuzuka, R., Maemura, S., Morita, H., Nawata, K., Fujita, D., Taniguchi, Y., Yamauchi, H., Yagi, H., and Kato, M.*, et al.* (2018). Impact of Pathogenic FBN1 Variant Types on the Progression of Aortic Disease in Patients With Marfan Syndrome. Circ Genom Precis Med *11*, e2058.

Taniguchi, Y., Takeda, N., Inuzuka, R., Matsubayashi, Y., Kato, S., Doi, T., Yagi, H., Yamauchi, H., Ando, M., Oshima, Y., and Tanaka, S. (2021). Impact of pathogenic FBN1 variant types on the development of severe scoliosis in patients with Marfan syndrome. J MED GENET.

Xu, S., Li, L., Fu, Y., Wang, X., Sun, H., Wang, J., Han, L., Wu, Z., Liu, Y., and Zhu, J.*, et al.* (2020). Increased frequency of FBN1 frameshift and nonsense mutations in Marfan syndrome patients with aortic dissection. Mol Genet Genomic Med *8*, e1041.
